# HDAC7 knockout mitigates astrocyte reactivity and neuroinflammation via the IRF3/cGAS/STING signaling pathway

**DOI:** 10.3389/fncel.2025.1683595

**Published:** 2025-10-28

**Authors:** Rui-zhu Yue, Xing Guo, Wenqiang Li, Chaokun Li, Linlin Shan

**Affiliations:** 1 College of Biological Sciences, China Agricultural University, Beijing, China; 2 Henan Key Laboratory of Biological Psychiatry, The Second Affiliated Hospital of Xinxiang Medical University, Xinxiang, China; 3 Department of Biochemistry and Molecular Biology, School of Basic Medical Sciences, Henan Medical University, Xinxiang, China

**Keywords:** HDAC7, astrocyte, inflammation, LPS, IRF3, cGAS, STING, C3

## Abstract

**Introduction:**

Astrocytes are parenchymal cells widely distributed throughout the brain. Beyond their essential functions in healthy tissue, astrocytes exhibit an evolutionarily conserved response to all forms of brain injury, termed astrocytic reactivity. Nevertheless, conceptual understanding of what astrocytic reactivity encompasses and its functional roles remains incomplete and occasionally contentious. Lipopolysaccharide (LPS) is widely used to induce neuroinflammation. In the current study, Histone deacetylase 7 (HDAC7) has been shown to ameliorate LPS-induced neuroinflammation and mitigate astrocytic reactivity.

**Methods:**

We overexpressed HDAC7 using viral vectors and generated primary astrocytes from Hdac7^*flox*/*flox*^ mice to achieve astrocyte-specific HDAC7 knockout. Subsequently, we assessed astrocytic reactivity and detected the expression of the Interferon regulatory factor 3 (IRF3)/cyclic GMP-AMP synthase (cGAS)/stimulator of interferon genes (STING) pathway.

**Results:**

HDAC7 has been implicated in inflammatory regulation, but its role in astrocyte reactivity and the underlying mechanisms remain unclear. Here, we demonstrate that HDAC7 deficiency attenuates LPS-induced astrogliosis by suppressing the cGAS/STING signaling axis. LPS stimulation induced robust upregulation of glial fibrillary acidic protein (GFAP), complement component 3 (C3), and pro-inflammatory cytokines (TNF-α, IL-6) in WT astrocytes, which was significantly blunted in HDAC7 knockout astrocytes. Conversely, lentiviral overexpression of HDAC7 in WT astrocytes exacerbated IRF3/cGAS/STING pathway activation, as validated by Western blot analysis showing upregulated cGAS, STING and IRF3 expression. Pharmacological activation of the STING pathway in astrocytes restored pro-inflammatory cytokine expression and reactive marker levels, indicating pathway dependence.

**Discussion:**

Our results delineate a novel HDAC7/IRF3/cGAS/STING signaling axis that governs astrocyte reactivity. This discovery provides a crucial cellular neurophysiological mechanism by which astrocytes integrate inflammatory signals and subsequently modulate the central nervous system microenvironment. Targeting HDAC7, therefore, represents a therapeutic strategy to mitigate neuroinflammation by specifically correcting this aberrant cell-physiological state of astrocytes, ultimately preserving neural circuit function.

## 1 Introduction

As the predominant glial subtype in the central nervous system (CNS), astrocytes are crucial for providing trophic and metabolic support to neurons, regulate synaptogenesis and neuroinflammatory responses, and contribute to blood-brain barrier (BBB) formation (Xiong et al., 2022). Reactive astrocytes are a state of astrocytes in the CNS that undergo morphological, physiological, and functional changes in response to injury or disease stimuli. They play a complex and dual role in pathological conditions. Specifically, when astrocytes differentiate into the A1 phenotype, reactive astrocytes become overactivated and release large amounts of pro-inflammatory cytokines (Liddelow et al., 2017). Excessive pro-inflammatory cytokines can further exacerbate the inflammatory response, leading to BBB, neuronal apoptosis, and neurological dysfunction. This process promotes the progression of neurodegenerative diseases, as excessive inflammation accelerates the death of nerve cells (Escartin et al., 2021; Hinkle et al., 2019; Liang et al., 2023).

This mechanism ensures immune quiescence during homeostasis and activates pro-inflammatory signaling in response to pathological stimulation. Under chronic neuroinflammation or infection, astrocytes adopt a neurotoxic A1 phenotype (Fang et al., 2022), marked by upregulation of complement proteins, pro-inflammatory cytokines (IL-6, TNF-α), and chemokines (CXCL10). This reactive astrogliosis can be induced by ATP/P2X7-mediated Ca^2+^ signaling or microglial IL-1α, TNF-α, and C1q. A1 astrocytes contribute to disease pathology via mechanisms such as oxidative stress in stroke (Sun et al., 2025; Wang et al., 2024), Excitatory amino acid transporter 2 (EAAT2) dysfunction in Amyotrophic Lateral Sclerosis (ALS) (Rosenblum et al., 2017), D-serine-mediated N-Methyl-D-aspartic acid (NMDA) potentiation in chronic pain (Sethuraman et al., 2009), C3-driven synapse loss in Alzheimer’s Disease (AD) (Wen et al., 2024), and neurotoxin release in HIV-associated neurocognitive disorders (HAND) (Lun et al., 2023). In contrast, A2 astrocytes exert neuroprotective roles through Brain-derived neurotrophic factor/Glial-cell-line-derived neurotrophic factor (BDNF/GDNF) secretion and Aquaporin-4 (AQP4) -mediated edema regulation (Liddelow et al., 2017).

Class IIa histone deacetylases (HDACs 4, 5, 7, 9) modulate inflammation through epigenetic gene regulation and deacetylation of non-histone substrates in cancer (Shakespear et al., 2011). We investigated HDAC inhibition, which confers anti-inflammatory properties, in models of colitis and inflammation-induced tumorigenesis. The treatment demonstrated significant efficacy in suppressing both inflammation and tumor development (Glauben et al., 2009). HDAC7 overexpressed in astrocyte and was shown to promote nuclear factor kappa-light-chain-enhancer of activated B (NF-κB) activation and upregulate pro-inflammatory genes in LPS-induced mice (Ye et al., 2022). The IRF3/cGAS/STING pathway activates antiviral inflammation through type-I interferon (IFN-I) and NF-κB signaling (Balka et al., 2020). Recent studies implicate the cGAS-STING pathway in neuroinflammation (Paul et al., 2021), where cytosolic DNA sensing triggers Cyclic GMP-AMP (cGAMP) synthesis, STING activation, and IFN-I induction—observed in Aβ-mediated microglial activation in AD (Govindarajulu et al., 2023), antiviral responses in viral encephalitis (Losarwar et al., 2025), and STING knockout suppressing astrocyte proliferation (Zhang et al., 2020). Microglial STING activation contributes to neuroinflammation and neurodegeneration in α-synucleinopathies, including Parkinson’s disease (PD) (Hinkle et al., 2022). The emergence of reactive astrocytes occurs concomitantly with neuroinflammation.

To date, no studies have demonstrated the involvement of HDACs in the IRF3/cGAS/STING system-induced inflammatory and reactive states of astrocytes. While our previous work established that LPS upregulates HDAC7 in astrocytes to drive pro-inflammatory responses via NF-κB activation, the precise mechanism by which LPS signaling promotes the polarization of astrocytes toward the detrimental A1 reactive state remains unknown. Here, we investigate whether the IRF3/cGAS/STING pathway serves as the critical link connecting LPS-induced HDAC7 upregulation to A1 astrocyte polarization.

## 2 Materials and methods

### 2.1 Animals

Hdac7^*flox*/*flox*^ mice were obtained from GenPharmatech. Adult C57BL/6 mice were purchased from Changzhou Cavens Laboratory Animal Co., Ltd (Changzhou, China). Hdac7^*flox*/*flox*^ mice (4 months of age) were individually housed in ventilated cages (IVC; 4 mice per cage) under controlled temperature (22 ± 1 °C) with a standardized 12 h/12 h light/dark cycle. Food (standard rodent chow) and water were provided *ad libitum*. All animal procedures were conducted in compliance with the National Institutes of Health Guide for the Care and Use of Laboratory Animals and were carried out under a protocol approved by the Institutional Animal Care and Use Committee (IACUC) of Xinxiang Medical University.

### 2.2 Primary astrocyte culture

Newborn Hdac7^*flox*/*flox*^ mice (postnatal day 1–3) were selected, as cortical astrocytes are abundant and viable at this stage. Cortices were dissected under sterile conditions in a laminar flow hood. Meninges and hippocampi were carefully removed. Cortical tissues were transferred into dissociation solution (30 mL HBSS+330 μL HEPES+1.5 mL 10% Glucose) for enzymatic dissociation. An equal volume of 0.25% trypsin was added for 20 min digestion at 37 °C, terminated by adding 2 mL fetal bovine serum (FBS). The dissociated cell suspension was seeded onto poly-D-lysine (PDL)-coated culture plates and maintained for 7 days in culture medium (90% DMEM high-glucose+10% FBS+1% penicillin/streptomycin). After 7 days, cells were placed on an orbital shaker (37 °C, 1,000 rpm for 12 h). The supernatant containing microglia and oligodendrocyte precursors was discarded. Adherent astrocytes were trypsinized and re-seeded onto new 6-well culture plates at a density of 5 × 10^6^ cells/mL. Upon full adhesion, the cells were treated with 250 ng/mL LPS for 48 h before being harvested.

### 2.3 PCR

Genomic DNA extraction was performed using the Beyotime Mouse Genomic DNA Extraction Kit (D7283S). Freshly prepared digestion buffer (96 μL DNA Extraction Solution+4 μL Enzyme Mix per sample) was used to digest 0.2–1 cm tail tips, hair follicles with roots, or 10 μL saliva in 100 μL buffer (55 °C for 15 min → 95 °C for 5 min). Reactions were terminated with 100 μL Stop Solution, with samples stored at −20 °C/4 °C. For PCR amplification, 20 μL reaction mixtures were prepared on ice containing: 7.4 μL dd H2 O, 1 μL template DNA (2–20 ng/μL), 1.6 μL primer mix (Hdac7^*flox*/*flox*^ Forward: 5′-TCAGGAAGCCAGTACACCAGAA CTG-3′; Reverse: 5′-GGAAAGAGCTTGTGGGACGTCAC-3′ Wild type: 247 bp, Mutant: 352 bp), and 10 μL Easy-Load™ PCR Master Mix. After centrifugation, thermal cycling proceeded as: 95 °C 5 min (1 cycle); 10 cycles of [95 °C 20 sec → 60 °C 20 sec (Δ−0.5 °C/cycle) → 72 °C 50 sec]; 26 cycles of (95 °C 20 sec → 55 °C 20 sec → 72 °C 50 sec); final extension at 72 °C 5 min with temporary 4 °C hold. PCR products were directly analyzed by agarose gel electrophoresis. Critical precautions included: using sterile equipment, wearing gloves to prevent contamination, preparing digestion buffer fresh, and aliquoting samples for long-term storage to avoid freeze-thaw cycles (The results show in the Supplementary material).

### 2.4 Western blot

Primary astrocytes were lysed using RIPA buffer supplemented with protease and phosphatase inhibitors and subsequently harvested by cell scraping. The resulting lysates were sonicated on ice for 5 min, followed by centrifugation at 12,000 × *g* for 20 min at 4 °C to collect the supernatant. Protein concentration was determined with a BCA assay kit (Beyotime, Cat# P0009). Protein samples were denatured in loading buffer at 95 °C for 10 min and electrophoresed on 10% or 12.5% SDS-polyacrylamide gels at 100 V for approximately 90 min. Subsequently, proteins were transferred onto nitrocellulose (NC) membranes (Thermo Fisher Scientific, Cat# 26616) at 256 mA for 30 min (proteins < 30 kDa) or 120 min (proteins 30–180 kDa). The membranes were blocked with 5% non-fat milk in TBST for 1 h at room temperature, incubated with primary antibodies (see Table 1) overnight at 4 °C, and then washed three times (10 min each) with TBST. Thereafter, membranes were probed with HRP-conjugated secondary antibodies for 1 h at room temperature, followed by three additional washes with TBS. Protein bands were visualized using an enhanced chemiluminescence (ECL) detection system.

**TABLE 1:** Primers used for realtime PCR.

Genes	Forward (5′ to 3′)	Reverse (5′ to 3′)
GAPDH	TGTGAACGGATTTG GCCGTA	ACTGTGCCGTTGAAT TTGCC
HDAC7	GAACTCTTGAGCCCT TGGACA	GGTGTGCTGCTACT ACTGGG
C3	AAGCATCAACACACC CAACA	CTTGAGCTCCATTCGT GACA
C1Q	AAAGGCAATCCAGGCA ATATCA	TGGTTCTGGTATGGA CTCTCC
COX-2	GTCTGGTGCCTGGT CTGATGATG	TCTGATACTGGAACTGCT GGTTGAA
FKBP5	GGACTGGACAGTGC CAATGAGA	CGTTGTGCTCCTT CGCCTTC
GFAP	AGAAAGGTTGAATC GCTGGA	CGGCGATAG TCGTTA
GBP2	CCTGGTTCTGCTTGAC ACTGAG	TGCTGGTTGATGGTTC CTATGC
H2–D1	GGCTCCACAGATAC CTGAAGAAC	CAAGAGGCACCACC ACAGATG
H2-T23	CTCCTCCATCCACTG TCTCCAA	ACCTATGTGTCTCCTC CTCTTCAT
HSBP1	ATCCCCTGAGGGCAC ACTTA	GGAATGGTGATCTCCG CTGAC
IFN-γ	CGGCACAGTCATT GAAAGCC	TGCATCCTTTTTCG CCTTGC
IL-1α	CGCTTGAGTCGGC AAAGAAAT	CTTCCCGTTGCTT GACGTTG
IL-1β	GCAACTGTTCCTG AACTCAACT	ATCTTTTGGGGTC CGTCAACT
IL-6	TAGTCCTTCCTACCCCAA TTTCC	TTGGTCCTTAGCCACT CCTTC
iNOS	CCCTTCCGAAGTTTCTGG CAGCAGC	GGCTGTCAGAGCCTCGTGG CTTTGG
Lcn2	TGGCCCTGAGTGTC ATGTG	CTCTTGTAGCTCATAGAT GGTGC
PSMB8	CACCGCATTCCTGAGG TCCTT	GGAGTCCACAGCCAC GATGA
Serpina3n	ATTTGTCCCAATGTCT GCGAA	TGGCTATCTTGGCTATAA AGGGG
TNF-α	CCCTCACACTCAGATCA TCTTCT	GCTACGACGTGGG CTACAG
VIM	CGTCCACACGCACCTACAG	GGGGGATGAGGAAT AGAGGCT

### 2.5 Realtime PCR

Total RNA was extracted from cultured cells with pre-chilled TRIzol™ Reagent (Invitrogen, Cat# 15596026) according to the manufacturer’s instructions. Briefly, after removing the culture medium, cells were directly lysed in TRIzol. RNA was precipitated through sequential phase separation using chloroform, followed by isopropanol precipitation and washing with 75% ethanol. The RNA pellet was collected by centrifugation at 12,000 × *g* for 15 min at 4 °C. RNA concentration and purity were measured using a NanoDrop 2000 spectrophotometer (Thermo Fisher Scientific), and samples with A260/A280 ratios between 1.8 and 2.0 were used for subsequent experiments. First-strand cDNA was synthesized from total RNA using the PrimeScript™ RT Master Mix (Takara Bio, Cat# RR047A). The process included two steps: (1) genomic DNA removal with gDNA Eraser at 25 °C for 5 min, and (2) reverse transcription at 37 °C for 15 min, followed by enzyme inactivation at 85 °C for 5 sec and cooling to 4 °C. Realtime PCR was performed on a Bio-Rad CFX96 Real-Time PCR System using 2× Real Star Fast SYBR qPCR Mix (Gene Star, Cat# A301-10). Each 10 μL reaction contained 5 μL of qPCR mix, 0.8 μL each of forward and reverse primers (10 μM), 2 μL of cDNA template, and 2.2 μL of nuclease-free water. The thermal cycling conditions were as follows: initial denaturation at 95 °C for 30 sec; 40 cycles of 95 °C for 5 sec and 60 °C for 30 sec; and melt curve analysis from 60 to 95 °C with incremental heating at 0.5 °C/sec. Threshold cycle (Cq) values were determined using Bio-Rad CFX Maestro™ software. Relative mRNA expression levels were calculated via the 2^–ΔΔCt^ method, with glyceraldehyde-3-phosphate dehydrogenase (GAPDH) serving as the endogenous reference gene. Primer sequences can are listed in Table 2.

**TABLE 2:** Primary antibodies used in this study.

Antibodies	Applications	Source	Catelogy
β-actin	WB: 1:2,000	Proteintech	Cat#66009
cGAS	WB: 1:1,000	Proteintech	Cat#29958
STING	WB: 1:1,000	CST	Cat#13647S
IRF3	WB: 1:1,000	Proteintech	Cat#66670
INOS	WB: 1:1,000	CST	Cat#13120S
COX-2	WB: 1:1,000	Abcam	Cat#ab15191
IL-6	WB: 1:1,000	CST	Cat#12912S
IL-1β	WB: 1:1,000	Proteintech	Cat#16806
GFAP	WB: 1:1,000	CST	Cat#3670S
HDAC7	WB: 1:1,000	Sigma-Aldrich	Cat# H2662

### 2.6 Immunofluorescence

Cells were fixed with 4% paraformaldehyde for 15–20 min to preserve cellular morphology. Permeabilization was carried out with 0.3% Triton X-100 (P0096, Beyotime) for 30 min to facilitate antibody access. Following this, blocking was performed with 5% BSA at room temperature for 1 h to minimize nonspecific binding. Subsequently, primary antibodies, diluted to their optimal working concentrations, were applied and incubated overnight at 4 °C to allow specific antigen binding. The next day, samples underwent three 5 min PBS washes to remove unbound primary antibodies. Fluorescently conjugated secondary antibodies were incubated for 1 h at 37 °C protected from light. After three additional 5 min PBS washes to remove excess secondary antibodies, nuclei were counterstained with DAPI for 5 min. Mounted specimens were imaged using fluorescence microscopy.

### 2.7 Flow cytometry

Prior to the initiation of the procedure, all necessary reagents were prepared, including 1X PBS, 4% formaldehyde, 100% methanol, and 0.5% BSA in PBS (stored at 4 °C). For fixation, adherent cells or tissues were dissociated into single-cell suspensions and centrifuged at 150–300 *g* for 5 min to pellet the cells. The cell pellet was resuspended in approximately 100 μL of 4% formaldehyde per 1 × 10^6^ cells and mixed thoroughly to dissociate aggregates and prevent cell clumping. Fixation was performed at room temperature (20–25 °C) for 15 min. After fixation, cells were washed 2–3 times with excess 1X PBS via centrifugation — care being taken to avoid excessive g-forces to prevent cell rupture — and finally resuspended in 0.5–1 mL of 1X PBS for immediate use or stored overnight at 4 °C. For permeabilization, fixed cells were gently resuspended, and prechilled 100% methanol was added dropwise to achieve a final concentration of 90%, followed by incubation on ice for at least 10 min. For immunostaining, an appropriate number of cells were aliquoted into 1 mL microcentrifuge tubes, washed 2–3 times with 1X PBS under gentle centrifugation, and incubated with 100 μL of primary antibody solution for 1 h at room temperature with occasional mixing. Cells were then washed again and incubated with 100 μL of secondary antibody solution for 1 h at room temperature protected from light. After a final wash, cells were resuspended in 200–500 μL of 1X PBS. Processed samples were analyzed using a flow cytometer. The purity of astrocytes was assessed by flow cytometry, and the representative gating strategy and quantification are presented in Supplementary Figure 2.

### 2.8 ELISA

Quantification of interleukin-6 (IL-6) and interleukin-1 beta (IL-1β) in lysates of primary astrocytes was performed using specific ELISA kits (MEIMIAN, #MM-0163M1 and #MM-0040M1, respectively) in strict adherence to the manufacturer’s protocol. The assay procedure involved the addition of 50 μL of standards or samples to the antibody-precoated wells, followed by a 30-min incubation at 37 °C. The wells were then aspirated and washed three times with wash buffer. Subsequently, 100 μL of horseradish peroxidase (HRP)-conjugated detection antibody was added to each well, and the plate was incubated for a further 30 min at 37 °C. After a second wash cycle, color development was initiated by sequential addition of 50 μL of substrate solutions A and B, with a 15-min incubation at 37 °C in the dark. The enzymatic reaction was stopped by adding 50 μL of stop solution per well. Absorbance was measured immediately at 450 nm, and sample cytokine concentrations were determined by interpolation from a standard curve calibrated with recombinant proteins.

### 2.9 Statistical analysis

Statistical analyses were performed with Graphpad Prism 10 (LaJolla, CA, USA). Unpaired Student’s *t*-test was employed to identify difference between the two groups. One-way or two-way analysis of variance (ANOVA) followed by Tukey’s multiple comparisons test was employed to identify differences among three or more groups as indicated in the figure legends. The statistical significance levels were set at *p* < 0.05 (*), *p* < 0.01 (**), *p* < 0.001 (***), with a confidence interval of 95%. All data were expressed as mean ± SD.

## 3 Results

### 3.1 LPS triggers astrocyte reactivity and neuroinflammatory responses

**FIGURE 1:**
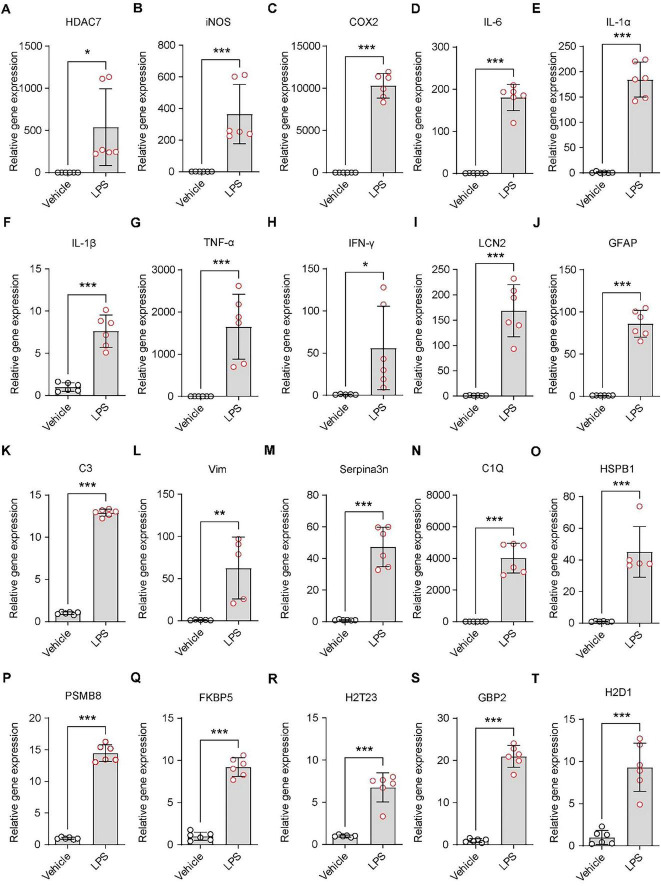
LPS triggers astrocyte reactivity and neuroinflammatory responses. **(A–T)** Quantitative PCR analysis of mRNA expression levels in primary astrocytes treated with 250 ng/mL LPS. **(A)** HDAC7, **(B)** iNOS, **(C)** COX-2, **(D)** IL-6, **(E)** IL-1α, **(F)** IL-1β, **(G)** TNF-α, **(H)** IFN-γ, **(I)** LCN2, **(J)** GFAP **(K)** C3, **(L)** Vim, **(M)** Serpina3n, **(N)** C1Q, **(O)** HSBP1, **(P)** PSMB8, **(Q)** FKBP5, **(R)** H2-T23, **(S)** GBP2, and **(T)** H2-D1, *n* = 5–6. Data are presented as mean ± SD. Statistical significance was determined using unpaired *t*-test. ***p < 0.001, **p < 0.01, *p < 0.05.

Following treatment of primary astrocytes with 250 ng/mL LPS for 48 h, we detected HDAC7 expression to validate our previous findings (Ye et al., 2022), confirming that LPS induces significant upregulation of HDAC7 (Figure 1A). iNOS, an enzyme increased during inflammation, primarily catalyzes nitric oxide (NO) production (Hibbs et al., 1988; Moncada et al., 1991). NO exhibits cytotoxicity by damaging DNA, proteins, and cell membranes (Xia et al., 2010). Concurrently, NO upregulates other pro-inflammatory factors (e.g., IL-1β, TNF-α), establishing a pro-inflammatory positive feedback loop. LPS markedly increased iNOS expression (Figure 1B), accompanied by elevated IL-1β (Figure 1F) and TNF-α expression (Figure 1G). COX-2, a key enzyme in prostaglandin synthesis (Kujubu et al., 1991), showed significant upregulation in astrocytes under sustained LPS stimulation (Figure 1C). A pronounced increase in IL-6 further indicated progressive enhancement of astrocytic inflammatory responses (Figure 1D). Peripheral administration of LPS is known to trigger a central inflammatory response and robustly stimulate IL-1α expression from astrocytes (Figure 1E). While inducing substantial elevations in inflammatory gene (iNOS, COX-2, IL-1β, TNF-α), LPS also significantly upregulated IFN-γ expression (Figure 1H). LCN2, initially identified in neutrophils and widely expressed in various cells (epithelial, immune, neuronal), participates in inflammatory and immune responses (Kjeldsen et al., 1994). It demonstrated marked upregulation following stimulation by inflammatory cytokines (IL-1β, TNF-α, IFN-γ) (Figure 1I). Elevated LCN2 further amplified TNF-α and IL-6 release. GFAP, a specific astrocytic marker critical for maintaining cellular structure, function, and CNS homeostasis (Bignami et al., 1972), showed significant overexpression (Figure 1J) in activated “reactive astrocytes,” accompanied by cellular hypertrophy and increased processes. Astrocytic complement C3 expression, observed in various pathological conditions, was substantially increased by LPS (Figure 1K). Vimentin (Vim), a type III intermediate filament protein, is predominantly expressed in radial glia and immature astrocytes during CNS development, later replaced by GFAP in adults. In mature brain tissue, Vim persists in subependymal immature astrocytes and cerebellar Bergmann glia (Hockfield and McKay, 1985). LPS induced Vim upregulation in astrocytes (Figure 1L). Serpina3n, an acute-phase protein with anti-inflammatory effects in early cerebral ischemia by suppressing microglial overactivation (Zhang J. et al., 2021), exhibited sustained overexpression under LPS-induced model (Figure 1M), potentially promoting pro-inflammatory A1 astrocyte maintenance and impairing neural repair. Under neuroinflammatory conditions, inflammatory microglia secrete IL-1α, TNF, and C1q to induce astrocytic activation into neurotoxic states. Our study confirmed LPS-induced IL-1α and TNF upregulation, with concurrent C1Q elevation (Figure 1N), indicating significant neuroinflammation and A1 reactive astrocyte transformation. HSBP1, a reactive astrocyte marker, was upregulated by LPS (Figure 1O). LPS-induced PSMB8 overexpression (Figure 1P) triggered endoplasmic reticulum stress and excessive inflammatory cytokine release (IFN-γ, IL-6). While astrocytes normally support myelination, LPS-induced A1 transformation led to FKBP5 overexpression (Figure 1Q), inhibiting remyelination. Classic A1 astrocyte markers H2-T23, GBP2, and H2-D1 all showed significant LPS-induced upregulation (Figures 1R–T).

### 3.2 Overexpression of HDAC7 activates IRF3/cGAS/STING and induces astrocyte activation and inflammatory responses

**FIGURE 2:**
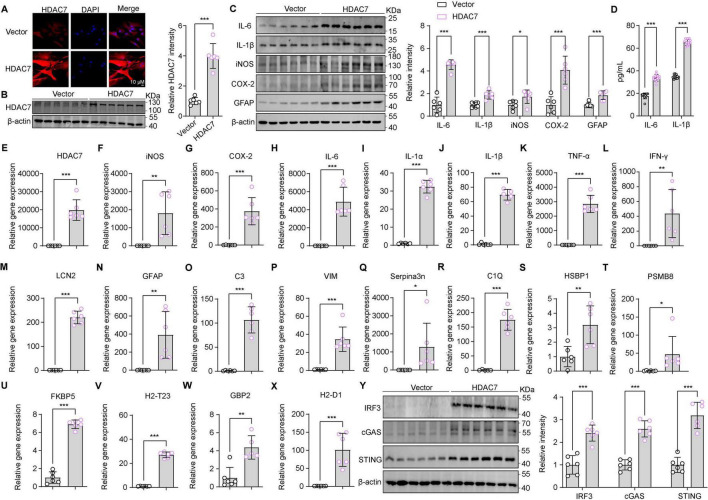
Overexpression of HDAC7 activates IRF3/cGAS/STING and induces astrocyte activation and inflammatory responses. **(A)** Representative immunostaining images of HDAC7 and DAPI in astrocyte (Scale Bar = 10 μm). **(B)** Representative Western blot images for HDAC7 and β-actin, and the corresponding quantitative data, *n* = 6. **(C)** Representative Western blot images of IL-6, IL-1β, iNOS, COX-2, GFAP and β-actin, with quantification of band intensities indicating the relative protein expression levels of IL-6, IL-1β, iNOS, COX-2 and GFAP, *n* = 6. **(D)** IL-6 and IL-1β concentrations were measured by ELISA, *n* = 12. **(E–X)** Quantitative PCR analysis of mRNA expression levels of various inflammatory and oxidative stress markers including **(E)** HDAC7, **(F)** iNOS, **(G)** COX-2, **(H)** IL-6, **(I)** IL-1α, **(J)** IL-1β, **(K)** TNF-α, **(L)** IFN-γ, **(M)** LCN2, **(N)** GFAP, **(O)** C3, **(P)** Vim, **(Q)** Serpina3n, **(R)** C1Q, **(S)** HSBP1, **(T)** PSMB8, **(U)** FKBP5, **(V)** H2-T23, **(W)** GBP2, and **(X)** H2-D1 in primary astrocyte infected with AAV-GfaABCD-GFP-WARE and AAV-GfaABCD-HDAC7-GFP-WARE, *n* = 5–6. **(Y)** Representative Western blot images showing the expression of IRF3, cGAS, STING, and β-actin, with quantification of band intensities indicating the relative expression levels of IRF3, cGAS, and STING, *n* = 6. Data are presented as mean ± SD. Data are presented as mean ± SD. Statistical significance was determined using unpaired *t*-test. ***p < 0.001, **p < 0.01, *p < 0.05.

Building on our previous finding that LPS upregulates astrocytic HDAC7—which enhances IKKα/β acetylation, activates NF-κB, and drives neuroinflammation—we next questioned whether HDAC7 overexpression alone is sufficient to induce astrocyte activation (Ye et al., 2022). Next, we identified whether HDAC7 overexpression can induce astrocyte activation. Primary mouse astrocytes transduced with LV-GfaABC1D-HDAC7-GFP-WPRE showed substantial upregulation of HDAC7 at both the mRNA and protein levels (Figures 2A, B, E). This was accompanied by a pronounced increase in the expression of key pro-inflammatory mediators—iNOS, COX-2, IL-6, and IL-1β are highly increased in protein and mRNA (Figures 2C, F–H, J). Notably, the elevated levels of IL-6 and IL-1β were further confirmed by ELISA (Figure 2D), demonstrating a consistent and significant upregulation. Reactive astrocyte marker GFAP (Figure 2N), additional cytokines IL-1α, TNF-α and IFN-γ (Figures 2I, K, L) were also elevated. Notably, HDAC7 overexpression upregulated both general (LCN2, GFAP) and A1-specific (C3, C1Q, GBP2) reactive astrocyte markers (Figures 2M–X), establishing HDAC7 as a key driver of A1 astrocyte transformation. Importantly, HDAC7 overexpression in astrocytes also significantly activated components of the STING pathway, including IRF3, cGAS, and STING (Figure 2Y).

### 3.3 HDAC7 deletion inhibits IRF3/cGAS/STING and ameliorates reactive astrogliosis and neuroinflammatory responses

**FIGURE 3:**
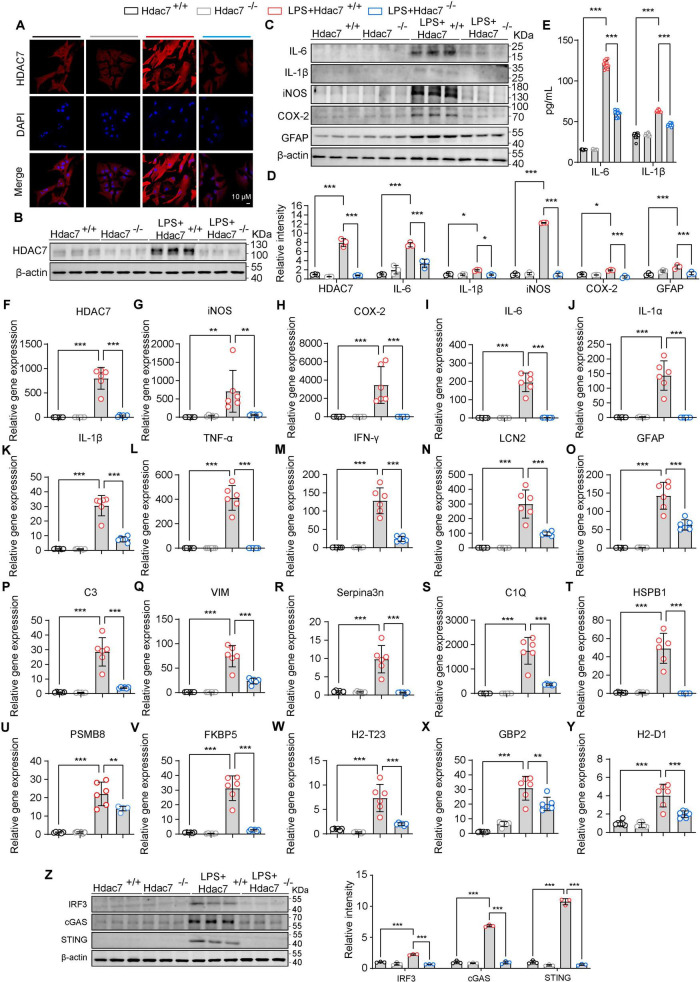
HDAC7 deletion inhibits IRF3/cGAS/STING and ameliorates reactive astrogliosis and neuroinflammatory responses. **(A)** Representative immunostaining images of HDAC7 and DAPI in astrocyte (Scale Bar = 10 μm). **(B)** Representative Western blot images of HDAC7 and β-actin, *n* = 3. **(C)** Representative Western blot images of IL-6, IL-1β, iNOS, COX-2, GFAP and β-actin, *n* = 3. **(D)** Quantification of HDAC7, IL-6, IL-1β, iNOS, COX-2, GFAP protein expression levels normalized to β-actin. **(E)** IL-6 and IL-1β concentrations were measured by ELISA, *n* = 12. **(F–Y)** Quantitative PCR analysis of mRNA expression levels of various inflammatory and reactive astrocyte markers including **(F)** HDAC7, **(G)** iNOS, **(H)** COX-2, **(I)** IL-6, **(J)** IL-1α, **(K)** IL-1β, **(L)** TNF-α, **(M)** IFN-γ, **(N)** LCN2, **(O)** GFAP, **(P)** C3, **(Q)** Vim, **(R)** Serpina3n, **(S)** C1Q, **(T)** HSBP1, **(U)** PSMB8, **(V)** FKBP5, **(W)** H2-T23, **(X)** GBP2, and **(Y)** H2-D1, *n* = 5–6. **(Z)** Representative Western blot images of IRF3, cGAS, STING, and β-actin, with quantification of band intensities indicating the relative expression levels of IRF3, cGAS, and STING. The primary astrocyte transfected with pLenti-EF1-P2A-puro-CMV-3xFLAG-WPRE (HDAC7^+/+^) and pLenti-EF1-P2A-puro-CMV-Cre-3xFLAG-WPRE (HDAC7^–/–^). And We treated primary astrocytes with 250 ng/mL LPS, *n* = 3. Data are presented as mean ± SD. Statistical significance was determined using one-way ANOVA with Tukey’s *post-hoc* test. ***p < 0.001, **p < 0.01, *p < 0.05.

Building upon our finding that HDAC7 upregulation drives astrocyte transformation into reactive A1 astrocytes, we generated Hdac7^*flox*/*flox*^ mice and isolated primary astrocytes from neonates. Cells were transduced with pLenti-EF1-P2A-puro-CMV-Cre-3xFLAG-WPRE to knockout HDAC7, assessing whether this attenuates LPS-induced inflammation and reactivity. Western blot analysis and immunofluorescence confirmed a significant reduction in HDAC7 expression following viral infection (Figures 3A, B, F). Additionally, we monitored key inflammatory markers, including cytokines (IL-6, IL-1β) and enzymes (iNOS, COX-2). HDAC7 knockout significantly attenuated the LPS-induced upregulation of these factors, as demonstrated by both western blot, Elisa and quantitative PCR analyses (Figures 3C, D, G–K). The reduced expression levels of IL-6 and IL-1β were further validated by ELISA in primary astrocytes, confirming consistent suppression following HDAC7 knockout (Figure 3E). Finally, we examined the effect of HDAC7 knockout on reactive astrocytes and found it significantly downregulated both general (LCN2, GFAP) and A1-specific (C3, C1Q, GBP2) markers (Figures 3L–Y), confirming its inhibitory role in A1 astrocyte polarization. Additionally, HDAC7 knockout suppressed LPS-induced activation of the cGAS/STING pathway (Figure 3Z). Collectively, these results indicate that HDAC7 deficiency prevents astrocytes from transforming into A1 reactive astrocytes.

### 3.4 HDAC7 deletion mitigates STING pathway-driven reactive astrogliosis and neuroinflammatory responses

**FIGURE 4:**
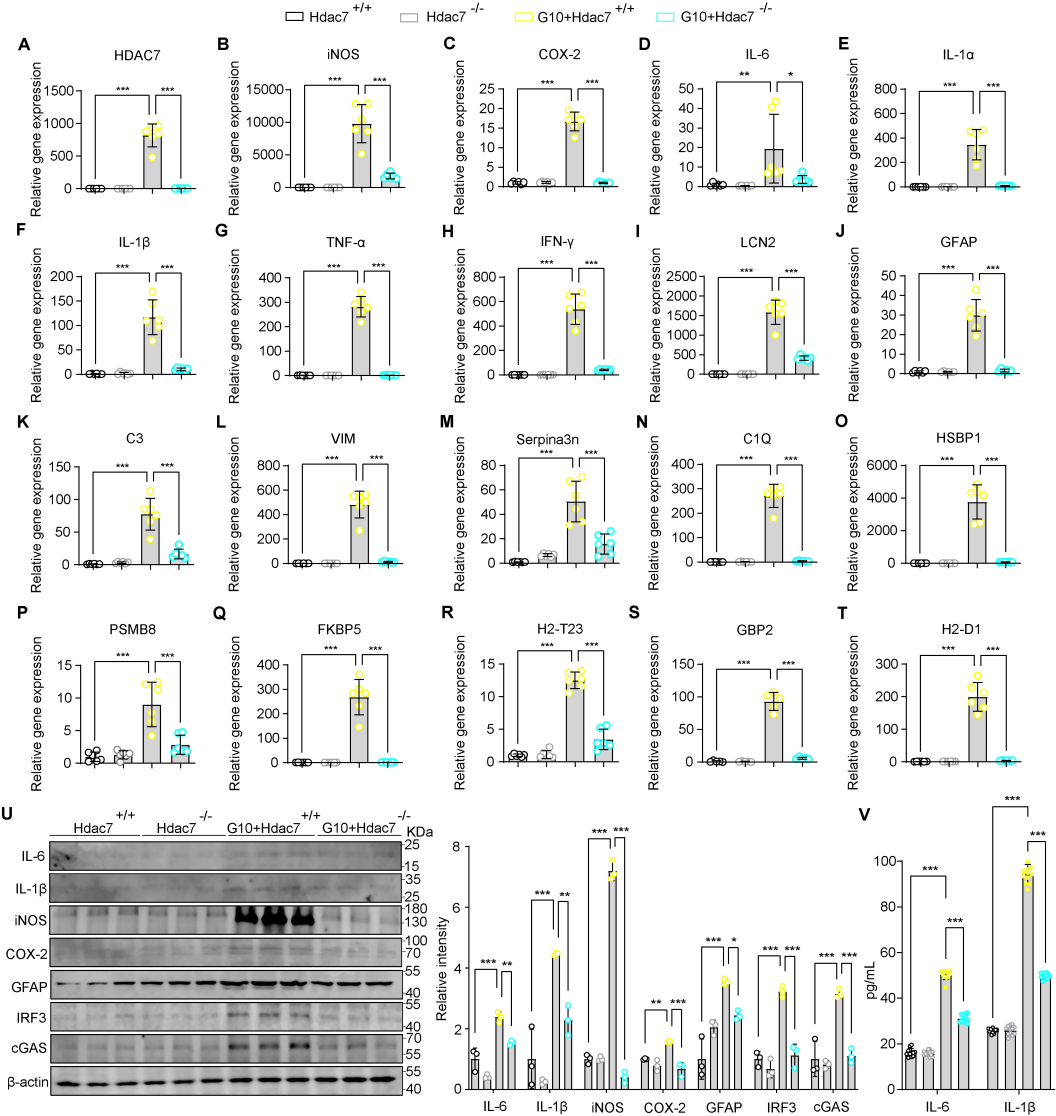
HDAC7 deletion mitigates STING pathway-driven reactive astrogliosis and neuroinflammatory responses. **(A–T)** Quantitative PCR analysis of mRNA expression levels of various inflammatory and oxidative stress markers including **(A)** HDAC7, **(B)** iNOS, **(C)** COX-2, **(D)** IL-6, **(E)** IL-1α, **(F)** IL-1β, **(G)** TNF-α, **(H)** IFN-γ, **(I)** LCN2, **(J)** GFAP **(K)** C3, **(L)** Vim, **(M)** Serpina3n, **(N)** C1Q, **(O)** HSBP1, **(P)** PSMB8, **(Q)** FKBP5, **(R)** H2-T23, **(S)** GBP2, and **(T)** H2-D1. The primary astrocyte transfected with pLenti-EF1-P2A-puro-CMV-3xFLAG-WPRE (HDAC7^+/+^) and pLenti-EF1-P2A-puro-CMV-Cre- 3xFLAG-WPRE (HDAC7^–/–^). And We treated primary astrocytes with 10 μM G10 (as the agonist of sting), *n* = 5–6. **(U)** Representative Western blot images of IL-6, IL-1β, iNOS, COX-2, GFAP, IRF3, cGAS, and β-actin, with quantification of band intensities indicating the relative expression levels, *n* = 3. **(V)** IL-6 and IL-1β concentrations were measured by ELISA, *n* = 12. Data are presented as mean ± SD. Statistical significance was determined using one-way ANOVA with Tukey’s *post-hoc* test. ***p < 0.001, **p < 0.01, *p < 0.05.

Building upon our finding that HDAC7 upregulation drives astrocyte transformation into reactive A1 astrocytes. Next, to assess whether HDAC knockout can attenuates LPS-induced inflammation and reactivity, primary astrocyte derived from Hdac7^*flox*/*flox*^ mice were transduced with G10 (a specific STING agonist). We investigated whether HDAC7 knockout could suppress astrocyte toxicity and reactive phenotypes. Initially, we assessed the expression levels of key inflammatory mediators at both protein and mRNA levels. The results showed significant reductions in IL-6, IL-1β, iNOS, and COX-2 (Figures 4B–D, F, U). Notably, the decreased expression of IL-6 and IL-1β was further confirmed by ELISA, consistent with the preceding data (Figure 4V). We also detected the reactive astrocyte activation marker GFAP and found it was also significantly reduced (Figure 4J). We detected the expression of IRF3 and cGAS and found significant decreases (Figure 4U), indicating that HDAC7 knockout inhibits the IRF3/cGAS/STING pathway. Next, we detected HDAC7 mRNA expression and found a significant decrease, suggesting that activating the STING pathway to induce inflammatory factor release also stimulates HDAC7 overexpression (Figure 4A). We also detected other relevant inflammatory factors, such as IL-1α (Figure 4E), TNF-α (Figure 4G), and IFN-γ (Figure 4H), and found that these inflammatory factors also decreased significantly, indicating that HDAC7 knockout can reduce the increased release of inflammatory factors caused by STING pathway activation. Finally, we verified whether HDAC7 knockout could inhibit the activation of reactive astrocytes and the high expression of A1 astrocyte markers after STING pathway activation. We found that reactive astrocyte markers, including LCN2 (Figure 4I), HSBP1 (Figure 4O), Serpina3n (Figure 4M), VIM (Figure 4L), and GFAP (Figure 4J) were significantly decreased; while established A1 astrocyte markers such as C3 (Figure 4K), C1Q (Figure 4N), PSMB8 (Figure 4P), FKBP5 (Figure 4Q), H2-T23 (Figure 4R), GBP2 (Figure 4S), and H2-D1 (Figure 4T) also showed significant reductions.

## 4 Discussion

This study systematically reveals the critical regulatory role of HDAC7 in LPS-induced A1 astrocyte activation and neuroinflammation, and for the first time demonstrates that HDAC7 drives neurotoxic astrocyte polarization by activating the IRF3/cGAS/STING signaling pathway, as validated by genetic knockout models showing the significant inhibitory effect of HDAC7 deficiency on neuroinflammation.

Glial cells are key components of the central nervous system with diverse and complex functions. Under physiological conditions, astrocytes interact with neurons through the “tripartite synapse” structure, directly regulating synaptic transmission and plasticity. They are responsible for maintaining extracellular ion/transmitter homeostasis and providing neurotrophic support. Meanwhile, microglia, as resident immune cells, play a central role in immune surveillance (Hanslik et al., 2021; Segarra et al., 2019; Vecino et al., 2016). A1 astrocyte activation is a common feature in pathological processes such as neurodegenerative diseases and brain injury (Hinkle et al., 2019); their overactivation can exacerbate neuronal damage by releasing complement proteins, pro-inflammatory cytokines, and neurotoxins (Yun et al., 2018). Elucidating the intrinsic mechanisms regulating neurotoxic versus neuroprotective astrocyte phenotypes remains a current research priority. Evidence indicates that the previously simplified A1: neurotoxic astrocyte and A2: neuroprotective astrocyte classification can be further subdivided into distinct subpopulations defined by proliferative capacity and differential gene expression profiles (Batiuk et al., 2020). Neuroprotective astrocytes act via compartmentalized cyclic adenosine monophosphate (cAMP) produced by soluble adenylyl cyclase. This regulation restrains microglial activation, leading to the suppression of downstream neurotoxic astrocyte induction (Cameron et al., 2024). Some studies also indicate that microglia play pivotal roles in the nervous system. Pharmacological or genetic approaches can induce robust adult neurogenesis (Dräger et al., 2022; Willis et al., 2020; Zhang Y. et al., 2021). Evidence further demonstrates that activated microglia release multiple inflammatory factors including IL-6, IL-1α, and TNF which drive astrocyte transformation into A1 astrocytes, thereby exacerbating severe neuroinflammatory responses in the nervous system (Liddelow et al., 2017). LPS-induced systemic inflammation leads to the generation of reactive astrocytes. The levels of inflammatory factors in astrocytes are significantly increased; it also exacerbates the activation of microglia, the disruption of the BBB, the infiltration of peripheral immune cells, neuronal dysfunction, and the depressive behaviors of mice (Guo et al., 2024). Under pathological conditions, factors such as neuroinflammation can activate microglia, which in turn drives the transformation of astrocytes into a neurotoxic A1-reactive phenotype. These reactive astrocytes release large amounts of pro-inflammatory factors, compromising BBB integrity and triggering neuronal apoptosis, thereby accelerating the progression of neurodegenerative diseases. In this process, molecules such as HDAC7 play a crucial role in glial cell activation and inflammatory responses by modulating key signaling pathways like IRF3/cGAS/STING, making them potential therapeutic targets for intervening in neuroinflammation and related diseases.

Therefore, the current discussions on A1 astrocytes remain inconclusive and are still a subject of debate. However, this study primarily focuses on elucidating the transformation mechanisms of normal astrocytes into A1-type astrocytes.

HDACs play a crucial role in modulating astrocyte function and neuroinflammation (Beurel, 2011; Manengu et al., 2024). In the context of neuroinflammation, HDACs modulate astrocyte activation, driving these cells toward a reactive phenotype marked by heightened generation of pro-inflammatory cytokines and chemokines (Wang et al., 2023). This astrocyte activation can exacerbate the inflammatory response and contribute to the compromise of the BBB (Kim et al., 2022). Targeting HDACs represents a potential therapeutic strategy to modulate astrocyte-mediated neuroinflammation and mitigate the associated neurodegenerative processes (Cai et al., 2022). Overexpression of Sirt1 reduces the reactivity of astrocytes, improves neurological dysfunction and improves neuron activity (Zhang et al., 2019, 2022). Additionally, HDACs may influence the crosstalk between astrocytes and other glial cells, such as microglia, further amplifying neuroinflammatory signaling (Villarreal et al., 2021). Our research found that LPS stimulation significantly upregulates HDAC7 expression in astrocytes, and HDAC7 overexpression further enhances the release of A1 markers and pro-inflammatory factors, suggesting HDAC7 is a key molecule linking PAMPs stimulation to the cytotoxic astrocyte phenotype.

Activation of the cGAS–STING axis orchestrates a multifaceted cellular program that encompasses senescence, autophagy, selective mRNA translation, and robust interferon-driven immunity (Wu et al., 2024). The cGAS/STING pathway is a central signaling axis for cytosolic DNA sensing. It is indispensable for mounting antiviral defenses, yet its dysregulation fuels autoimmune pathology and drives neuroinflammatory cascades (Oduro et al., 2022). This study found that HDAC7 overexpression significantly activates the protein expression of IRF3, cGAS, and STING, whereas HDAC7 deficiency reduces LPS or G10-induced inflammatory responses by inhibiting this pathway. It is noteworthy that STING activation can feedback to upregulate HDAC7 mRNA levels, suggesting a potential positive feedback regulatory loop between HDAC7 and the cGAS/STING pathway, which jointly sustains the chronicity of neuroinflammation. HDAC7 knockout mouse models constructed using CRISPR/Cas9 show that HDAC7 deficiency significantly inhibits LPS-induced astrocyte hypertrophy, GFAP expression, and upregulation of A1 markers, while concurrently reducing levels of pro-inflammatory factors such as IL-1β and TNF-α in brain tissue.

Under neuroinflammatory stress, LCN2 is rapidly synthesized and secreted by activated microglia and reactive astrocytes, propagating a cytotoxic milieu that culminates in widespread neuronal apoptosis. We demonstrate that LCN2 secretion from reactive astrocytes is triggered by LPS as an inflammatory stressor. Studies indicate that inhibiting NF-κB activation downregulates astrocytic LCN2 even under inflammatory stress (Jung et al., 2023). Our findings show that suppressing HDAC7 overexpression in astrocytes ameliorates LCN2 elevation, concurrently validating our prior research that NF-κB pathway activation enhances inflammatory factor release effects attenuated by TMP195-mediated inhibition of histone deacetylase function (Ye et al., 2022). Furthermore, this study establishes HDAC7 as a mediator of astrocyte transformation into A1-type reactive astrocytes. Mechanistically, this protective effect is closely associated with the inactivation of the IRF3/cGAS/STING pathway, indicating that HDAC7 may serve as a potential target for intervening in neuroinflammation-related diseases.

As research into the pathological mechanisms of neurodegenerative diseases advances, the neuroregulatory roles of HDACs and their specific inhibitors in these disorders are becoming increasingly elucidate. However, the physiological functions of HDAC7 in the nervous system, particularly its regulatory mechanisms in the activation of A1-reactive astrocyte, remain poorly understood and require further investigation. Based on our *in vitro* experimental results, we propose a central hypothesis: the HDAC7/cGAS-STING signaling pathway identified in this study may represent a key molecular mechanism driving the sustained activation of neurotoxic A1-reactive astrocytes. This mechanism could potentially explain the core pathological feature observed in neurodegenerative diseases such as Alzheimer’s disease namely, the persistent activation of astrocytes and exacerbated neuroinflammatory damage that are difficult to alleviate. Furthermore, future studies should focus on elucidating the regulatory patterns of this pathway *in vivo* using disease models, validating the synergistic neuroprotective effects of HDAC7-specific inhibitors and cGAS-STING pathway interventions, and clarifying the crosstalk between this pathway and other neuroinflammatory signaling cascades (Ye et al., 2022, 2025).

In the cGAS-STING signaling pathway, the initiation stage begins with the sensing of cytosolic double-stranded DNA. When exogenous DNA, such as viral DNA, or endogenous DNA, such as mitochondrial DNA leakage caused by genomic instability, is present in the cytoplasm, cGAS binds to and is activated by these dsDNA molecules. Subsequently, activated cGAS catalyzes the synthesis of the cyclic dinucleotide second messenger 2′3′-cGAMP from ATP and GTP. During the signal transduction phase, STING located on the endoplasmic reticulum, inducing conformational changes and dimerization of STING. The activated STING protein then translocates from the endoplasmic reticulum to the perinuclear region via the Golgi apparatus (Newman et al., 2024; West et al., 2015). Downstream STING activation involves the recruitment and activation of TBK1, leading to the phosphorylation and nuclear translocation of IRF3 and NF-κB, where they drive the expression of type I interferons (IFN-α/β) and inflammatory cytokines to trigger a broad innate immune response (Jiao et al., 2023). Previous studies have shown that class IIa HDACs can significantly activate IRF3 (Lu et al., 2024). Inspired by this finding, we investigated whether HDAC7 might also activate the cGAS-STING signaling pathway. In our study, we found that knockout of HDAC7 suppressed the cGAS-STING signaling pathway, thereby preventing excessive inflammatory responses in astrocytes.

HDAC7, a member of Class IIa histone deacetylases, exerts context-dependent and often complex regulatory roles in inflammation across different tissues and cell types. Its functions extend beyond histone modification to include deacetylation of transcription factors and cytoplasmic proteins, thereby influencing key inflammatory signaling pathways. In immune cells such as macrophages, HDAC7 is generally recognized as a positive regulator of pro-inflammatory responses. It enhances NF-κB activation and significantly upregulates the expression of pro-inflammatory cytokines such as TNF-α, IL-6, and IL-1β. The mechanism primarily relies on direct interaction with TRAF6 and TAK1 in the TLR4 signaling pathway, amplifying downstream MAPK and NF-κB activation without altering chromatin accessibility. Studies have shown that HDAC7 expression is significantly increased in LPS-induced inflammation models, and targeted degradation of HDAC7 using PROTAC technology effectively suppresses the release of multiple inflammatory cytokines (Kadier et al., 2024). The role of HDAC7 in inflammatory regulation remains incompletely understood; however, our findings indicate that it promotes the activation of the cyclic cGAS–STING pathway and exacerbates neuroinflammation. HDAC7 is a key regulator in TLR signaling. Its enzymatic activity can be rapidly activated by various TLR agonists in a MyD88 adaptor-dependent manner. This protein plays a dual role in inflammation: it regulates glycolysis in macrophages induced by low-dose LPS in a deacetylase-independent manner, while through its enzymatic activity—particularly via deacetylation of PKM2 and subsequent promotion of HIF-1α-mediated transcription—it enhances the production of pro-inflammatory cytokines such as IL-1β and CCL2. This functional divergence positions HDAC7 as a critical node linking inflammation and metabolic reprogramming, especially in low-grade chronic inflammation. Targeting HDAC7 enzymatic activity or its mediated protein interactions offers a potential new strategy for treating inflammation-related diseases (Ramnath et al., 2022). While current research has mainly focused on the strong pro-inflammatory role of HDAC7 in macrophages, studies also indicate that HDAC7 plays a significant pro-inflammatory role in LPS-induced inflammation by promoting the activation of astrocytes (Ye et al., 2022). Given its significant impact on inflammatory processes, HDAC7 has emerged as a potential therapeutic target in inflammatory and autoimmune diseases. Inhibition of HDAC7 has been shown to attenuate excessive immune activation in several experimental models, and our experiments further supplement its important role in ameliorating neuroinflammation, suggesting its utility in modulating inflammation-driven pathology.

Reactive astrogliosis, a hallmark of various neurological disorders, is characterized by the morphological and functional transformation of astrocytes in response to pathological stimuli. Several biomarkers have been identified to delineate reactive astrocytes (Liddelow et al., 2017). Activation of the IRF3/cGAS/STING pathway in microglia and astrocytes promotes the release of pro-inflammatory cytokines, chemokines, and interferons, exacerbating neuroinflammation (Decout et al., 2021).

It is noteworthy that HDAC7, belonging to class IIa HDACs, possesses tissue-specific expression and regulates non-histone substrates; these properties may confer lower systemic toxicity compared to broad-spectrum HDAC inhibitors, providing a theoretical basis for developing central nervous system-selective anti-inflammatory drugs. Although this study clarified the mechanism of HDAC7 action in *in vitro* astrocyte models, its role within the complex *in vivo* neural microenvironment requires further validation. For instance, whether HDAC7 indirectly influences neuroinflammation by regulating microglia-astrocyte interactions or exerts differential functions at distinct disease stages such as acute injury vs. chronic degeneration, needs to be elucidated by *in vivo* experiments. Furthermore, the interaction of HDAC7 with other inflammatory pathways, such as the NLRP3 inflammasome (Yao et al., 2022), and its impact on astrocyte differentiation toward the neuroprotective A2 phenotype, also warrants in-depth investigation. The functional plasticity of astrocytes makes them a potential therapeutic target for neurological diseases. Future research needs to leverage single-cell sequencing and spatial transcriptomics to decipher their heterogeneity and develop dual regulatory strategies, inhibiting the harmful A1 phenotype while preserving neuroprotective functions. In-depth research targeting the cGAS/STING pathway may provide novel insights for simultaneously intervening in neuroinflammation and glial scar formation.

This study elucidates that HDAC7 drives astrocyte transformation toward the neurotoxic A1 phenotype via the IRF3/cGAS/STING pathway, revealing the dual regulatory mechanism of HDAC7 in neuroinflammation involving simultaneous activation in IRF3/cGAS/STING pathways. The anti-inflammatory effect of HDAC7 knockout provides novel therapeutic strategies for inflammation-related brain diseases such as neurodegenerative disorders and stroke; targeting HDAC7 or its downstream pathways holds promise as a precision medicine approach for intervening in central nervous system inflammation.

## Data Availability

The original contributions presented in this study are included in this article/Supplementary material, further inquiries can be directed to the corresponding authors.

## References

[B1] Balka K. R. Louis C. Saunders T. L. Smith A. M. Calleja D. J. D’Silva D. B. (2020). TBK1 and IKKε act redundantly to mediate STING-Induced NF-κB responses in myeloid cells. *Cell Rep.* 31:107492. 10.1016/j.celrep.2020.03.056 32268090

[B2] Batiuk M. Y. Martirosyan A. Wahis J. de Vin F. Marneffe C. Kusserow C. (2020). Identification of region-specific astrocyte subtypes at single cell resolution. *Nat. Commun.* 11:1220. 10.1038/s41467-019-14198-8 32139688 PMC7058027

[B3] Beurel E. (2011). HDAC6 regulates LPS-tolerance in astrocytes. *PLoS One* 6:e25804. 10.1371/journal.pone.0025804 22022450 PMC3192131

[B4] Bignami A. Eng L. F. Dahl D. Uyeda C. T. (1972). Localization of the glial fibrillary acidic protein in astrocytes by immunofluorescence. *Brain Res.* 43 429–435. 10.1016/0006-8993(72)90398-8 4559710

[B5] Cai L. Zeng R. Huang Q. Liu X. Cao Z. Guo Q. (2022). Paeonol inhibits chronic constriction injury-induced astrocytic activation and neuroinflammation in rats via the HDAC/miR-15a pathway. *Drug Dev. Res.* 83 1758–1765. 10.1002/ddr.21993 36063531

[B6] Cameron E. G. Nahmou M. Toth A. B. Heo L. Tanasa B. Dalal R. (2024). A molecular switch for neuroprotective astrocyte reactivity. *Nature* 626 574–582. 10.1038/s41586-023-06935-3 38086421 PMC11384621

[B7] Decout A. Katz J. D. Venkatraman S. Ablasser A. (2021). The cGAS–STING pathway as a therapeutic target in inflammatory diseases. *Nat. Rev. Immunol.* 21 548–569. 10.1038/s41577-021-00524-z 33833439 PMC8029610

[B8] Dräger N. M. Sattler S. M. Huang C. T. Teter O. M. Leng K. Hashemi S. H. (2022). A CRISPRi/a platform in human iPSC-derived microglia uncovers regulators of disease states. *Nat. Neurosci.* 25 1149–1162. 10.1038/s41593-022-01131-4 35953545 PMC9448678

[B9] Escartin C. Galea E. Lakatos A. O’Callaghan J. P. Petzold G. C. Serrano-Pozo A. (2021). Reactive astrocyte nomenclature, definitions, and future directions. *Nat. Neurosci.* 24 312–325. 10.1038/s41593-020-00783-4 33589835 PMC8007081

[B10] Fang Y. Ding X. Zhang Y. Cai L. Ge Y. Ma K. (2022). Fluoxetine inhibited the activation of A1 reactive astrocyte in a mouse model of major depressive disorder through astrocytic 5-HT(2B)R/beta-arrestin2 pathway. *J. Neuroinflamm.* 19:23. 10.1186/s12974-022-02389-y 35093099 PMC8800238

[B11] Glauben R. Sonnenberg E. Zeitz M. Siegmund B. (2009). HDAC inhibitors in models of inflammation-related tumorigenesis. *Cancer Lett.* 280 154–159. 10.1016/j.canlet.2008.11.019 19101082

[B12] Govindarajulu M. Ramesh S. Beasley M. Lynn G. Wallace C. Labeau S. (2023). Role of cGAS–Sting signaling in Alzheimer’s disease. *Int. J. Mol. Sci.* 24:8151. 10.3390/ijms24098151 37175853 PMC10179704

[B13] Guo Q. Gobbo D. Zhao N. Zhang H. Awuku N. O. Liu Q. (2024). Adenosine triggers early astrocyte reactivity that provokes microglial responses and drives the pathogenesis of sepsis-associated encephalopathy in mice. *Nat. Commun.* 15:6340. 10.1038/s41467-024-50466-y 39068155 PMC11283516

[B14] Hanslik K. L. Marino K. M. Ulland T. K. (2021). Modulation of glial function in health, aging, and neurodegenerative disease. *Front. Cell. Neurosci.* 15:718324. 10.3389/fncel.2021.718324 34531726 PMC8439422

[B15] Hibbs J. B. Jr. Taintor R. R. Vavrin Z. Rachlin E. M. (1988). Nitric oxide: A cytotoxic activated macrophage effector molecule. *Biochem. Biophys. Res. Commun.* 157 87–94. 10.1016/s0006-291x(88)80015-9 3196352

[B16] Hinkle J. T. Dawson V. L. Dawson T. M. (2019). The A1 astrocyte paradigm: New avenues for pharmacological intervention in neurodegeneration. *Movement Disord.* 34 959–969. 10.1002/mds.27718 31136698 PMC6642014

[B17] Hinkle J. T. Patel J. Panicker N. Karuppagounder S. S. Biswas D. Belingon B. (2022). STING mediates neurodegeneration and neuroinflammation in nigrostriatal α-synucleinopathy. *Proc. Natl. Acad. Sci. U. S. A.* 119:e2118819119. 10.1073/pnas 35394877 PMC9169780

[B18] Hockfield S. R. D. M. McKay R. D. (1985). Identification of major cell classes in the developing mammalian nervous system. *J. Neurosci.* 5 3310–3328. 10.1523/JNEUROSCI.05-12-03310.1985 4078630 PMC6565218

[B19] Jiao P. Fan W. Ma X. Lin R. Zhao Y. Li Y. (2023). SARS-CoV-2 nonstructural protein 6 triggers endoplasmic reticulum stress-induced autophagy to degrade STING1. *Autophagy* 19 3113–3131. 10.1080/15548627.2023.2238579 37482689 PMC10621274

[B20] Jung B. K. Park Y. Yoon B. Bae J. S. Han S. W. Heo J. E. (2023). Reduced secretion of LCN2 (lipocalin 2) from reactive astrocytes through autophagic and proteasomal regulation alleviates inflammatory stress and neuronal damage. *Autophagy* 19 2296–2317. 10.1080/15548627.2023.2180202 36781380 PMC10351455

[B21] Kadier K. Niu T. Ding B. Chen B. Qi X. Chen D. (2024). PROTAC-Mediated HDAC7 protein degradation unveils its deacetylase-independent proinflammatory function in macrophages. *Adv. Sci.* 11:e2309459. 10.1002/advs.202309459 39049738 PMC11423193

[B22] Kim H. Leng K. Park J. Sorets A. G. Kim S. Shostak A. (2022). Reactive astrocytes transduce inflammation in a blood-brain barrier model through a TNF-STAT3 signaling axis and secretion of alpha 1-antichymotrypsin. *Nat. Commun.* 13:6581. 10.1038/s41467-022-34412-4 36323693 PMC9630454

[B23] Kjeldsen L. Bainton D. F. Sengelov H. Borregaard N. (1994). Identification of neutrophil gelatinase-associated lipocalin as a novel matrix protein of specific granules in human neutrophils. *Blood* 83 799–807. 10.1182/blood.V83.3.799.799 8298140

[B24] Kujubu D. A. Fletcher B. S. Varnum B. C. Lim R. W. Herschman H. R. (1991). TIS10, a phorbol ester tumor promoter-inducible mRNA from Swiss 3T3 cells, encodes a novel prostaglandin synthase/cyclooxygenase homologue. *J. Biol. Chem.* 266 12866–12872. 10.1016/S0021-9258(18)98774-0 1712772

[B25] Liang P. Zhang X. Zhang Y. Wu Y. Song Y. Wang X. (2023). Neurotoxic A1 astrocytes promote neuronal ferroptosis via CXCL10/CXCR3 axis in epilepsy. *Free Radical Biol. Med.* 195 329–342. 10.1016/j.freeradbiomed.2023.01.002 36610561

[B26] Liddelow S. A. Guttenplan K. A. Clarke L. E. Bennett F. C. Bohlen C. J. Schirmer L. (2017). Neurotoxic reactive astrocytes are induced by activated microglia. *Nature* 541 481–487. 10.1038/nature21029 28099414 PMC5404890

[B27] Losarwar S. Pancholi B. Babu R. Garabadu D. (2025). Mitochondria-dependent innate immunity: A potential therapeutic target in Flavivirus infection. *Int. Immunopharmacol.* 154:114551. 10.1016/j.intimp.2025.114551 40158432

[B28] Lu Y. Zhao Y. Gao C. Suresh S. Men J. Sawyers A. (2024). HDAC5 enhances IRF3 activation and is targeted for degradation by protein C6 from orthopoxviruses including Monkeypox virus and Variola virus. *Cell Rep.* 43:113788. 10.1016/j.celrep.2024.113788 38461415 PMC11650635

[B29] Lun J. Li Y. Gao X. Gong Z. Chen X. Zou J. (2023). Kynurenic acid blunts A1 astrocyte activation against neurodegeneration in HIV-associated neurocognitive disorders. *J. Neuroinflamm.* 20:87. 10.1186/s12974-023-02771-4 36997969 PMC10061717

[B30] Manengu C. Zhu C. H. Zhang G. D. Tian M. M. Lan X. B. Tao L. J. (2024). HDAC inhibitors as a potential therapy for chemotherapy-induced neuropathic pain. *Inflammopharmacology* 32 2153–2175. 10.1007/s10787-024-01488-x 38761314

[B31] Moncada S. R. M. J. Palmer R. M. L. Higgs E. (1991). Nitric oxide: Physiology, pathophysiology, and pharmacology. *Pharmacol. Rev.* 43 109–142. 10.1016/S0031-6997(25)06663-3 1852778

[B32] Newman L. E. Weiser Novak S. Rojas G. R. Tadepalle N. Schiavon C. R. Grotjahn D. A. (2024). Mitochondrial DNA replication stress triggers a pro-inflammatory endosomal pathway of nucleoid disposal. *Nat. Cell Biol.* 26 194–206. 10.1038/s41556-023-01343-1 38332353 PMC11026068

[B33] Oduro P. K. Zheng X. Wei J. Yang Y. Wang Y. Zhang H. (2022). The cGAS-STING signaling in cardiovascular and metabolic diseases: Future novel target option for pharmacotherapy. *Acta Pharm Sin B* 12 50–75. 10.1016/j.apsb.2021.05.011 35127372 PMC8799861

[B34] Paul B. D. Snyder S. H. Bohr V. A. (2021). Signaling by cGAS–STING in neurodegeneration, neuroinflammation, and aging. *Trends Neurosci.* 44 83–96. 10.1016/j.tins.2020.10.008 33187730 PMC8662531

[B35] Ramnath D. Das Gupta K. Wang Y. Abrol R. Curson J. E. B. Lim J. (2022). The histone deacetylase Hdac7 supports LPS-inducible glycolysis and Il-1β production in murine macrophages via distinct mechanisms. *J. Leukocyte Biol.* 111 327–336. 10.1002/jlb.2mr1021-260r 34811804

[B36] Rosenblum L. T. Shamamandri-Markandaiah S. Ghosh B. Foran E. Lepore A. C. Pasinelli P. (2017). Mutation of the caspase-3 cleavage site in the astroglial glutamate transporter EAAT2 delays disease progression and extends lifespan in the SOD1-G93A mouse model of ALS. *Exp. Neurol.* 292 145–153. 10.1016/j.expneurol.2017.03.014 28342750 PMC5433801

[B37] Segarra M. Aburto M. R. Hefendehl J. Acker-Palmer A. (2019). Neurovascular interactions in the nervous system. *Ann. Rev. Cell Dev. Biol.* 35 615–635. 10.1146/annurev-cellbio-100818-125142 31590587

[B38] Sethuraman R. Lee T. L. Tachibana S. (2009). D-serine regulation: A possible therapeutic approach for central nervous diseases and chronic pain. *Mini Rev. Med. Chem.* 9 813–819. 10.2174/138955709788452630 19519506

[B39] Shakespear M. R. Halili M. A. Irvine K. M. Fairlie D. P. Sweet M. J. (2011). Histone deacetylases as regulators of inflammation and immunity. *Trends Immunol.* 32 335–343. 10.1016/j.it.2011.04.001 21570914

[B40] Sun M. Song Y. Hu X. Zhang Z. Tan R. Cai Z. (2025). Leptin reduces LPS-induced A1 reactive astrocyte activation and inflammation via inhibiting p38-MAPK signaling pathway. *Glia* 73 25–37. 10.1002/glia.24611 39310943

[B41] Vecino E. Rodriguez F. D. Ruzafa N. Pereiro X. Sharma S. C. (2016). Glia–neuron interactions in the mammalian retina. *Prog. Retinal Eye Res.* 51 1–40. 10.1016/j.preteyeres.2015.06.003 26113209

[B42] Villarreal A. Vidos C. Monteverde Busso M. Cieri M. B. Ramos A. J. (2021). Pathological neuroinflammatory conversion of reactive astrocytes is induced by microglia and involves chromatin remodeling. *Front. Pharmacol.* 12:689346. 10.3389/fphar.2021.689346 34234677 PMC8255379

[B43] Wang H. K. Su Y. T. Ho Y. C. Lee Y. K. Chu T. H. Chen K. T. (2023). HDAC1 is involved in neuroinflammation and blood-brain barrier damage in stroke pathogenesis. *J. Inflamm. Res.* 16 4103–4116. 10.2147/jir.S416239 37745794 PMC10516226

[B44] Wang S. Pan Y. Zhang C. Zhao Y. Wang H. Ma H. (2024). Transcriptome analysis reveals dynamic microglial-induced A1 astrocyte reactivity via C3/C3aR/NF-κB signaling after ischemic stroke. *Mol. Neurobiol.* 61 10246–10270. 10.1007/s12035-024-04210-8 38713438

[B45] Wen L. Bi D. Shen Y. (2024). Complement-mediated synapse loss in Alzheimer’s disease: Mechanisms and involvement of risk factors. *Trends Neurosci.* 47 135–149. 10.1016/j.tins.2023.11.010 38129195

[B46] West A. P. Khoury-Hanold W. Staron M. Tal M. C. Pineda C. M. Lang S. M. (2015). Mitochondrial DNA stress primes the antiviral innate immune response. *Nature* 520 553–557. 10.1038/nature14156 25642965 PMC4409480

[B47] Willis E. F. MacDonald K. P. A. Nguyen Q. H. Garrido A. L. Gillespie E. R. Harley S. B. R. (2020). Repopulating microglia promote brain repair in an IL-6-Dependent manner. *Cell* 180 833–846 e16. 10.1016/j.cell.2020.02.013. 32142677

[B48] Wu Q. Leng X. Zhang Q. Zhu Y. Z. Zhou R. Liu Y. (2024). IRF3 activates RB to authorize cGAS-STING–induced senescence and mitigate liver fibrosis. *Sci. Adv.* 10:eadj2102. 10.1126/sciadv.adj2102 38416816 PMC10901380

[B49] Xia Z. Luo T. Liu H. M. Wang F. Xia Z. Y. Irwin M. G. (2010). L-arginine enhances nitrative stress and exacerbates tumor necrosis factor-α toxicity to human endothelial cells in culture: Prevention by propofol. *J. Cardiovasc. Pharmacol.* 55 358–367. 10.1097/FJC.0b013e3181d265a3 20125033

[B50] Xiong X.-Y. Tang Y. Yang Q.-W. (2022). Metabolic changes favor the activity and heterogeneity of reactive astrocytes. *Trends Endocrinol. Metab.* 33 390–400. 10.1016/j.tem.2022.03.001 35396164

[B51] Yao F. Jin Z. Zheng Z. Lv X. Ren L. Yang J. (2022). HDAC11 promotes both NLRP3/caspase-1/GSDMD and caspase-3/GSDME pathways causing pyroptosis via ERG in vascular endothelial cells. *Cell Death Discovery* 8:112. 10.1038/s41420-022-00906-9 35279683 PMC8918356

[B52] Ye J. Zhong S. Deng Y. Yao X. Liu Q. Wang J. Z. (2022). HDAC7 activates IKK/NF-kappaB signaling to regulate astrocyte-mediated inflammation. *Mol. Neurobiol.* 59 6141–6157. 10.1007/s12035-022-02965-6 35871708 PMC9309093

[B53] Ye J. Zhong S. Wan H. Guo X. Yao X. Liu Q. (2025). Upregulated astrocyte HDAC7 induces Alzheimer-like tau pathologies via deacetylating transcription factor-EB and inhibiting lysosome biogenesis. *Mol. Neurodegener.* 20:5. 10.1186/s13024-025-00796-2 39806423 PMC11727263

[B54] Yun S. P. Kam T. I. Panicker N. Kim S. Oh Y. Park J. S. (2018). Block of A1 astrocyte conversion by microglia is neuroprotective in models of Parkinson’s disease. *Nat. Med.* 24 931–938. 10.1038/s41591-018-0051-5 29892066 PMC6039259

[B55] Zhang D. Liu C. Li H. Jiao J. (2020). Deficiency of STING signaling in embryonic cerebral cortex leads to neurogenic abnormalities and autistic-like behaviors. *Adv. Sci.* 7:2002117. 10.1002/advs.202002117 33304758 PMC7710002

[B56] Zhang J. Rong P. Zhang L. He H. Zhou T. Fan Y. (2021). IL4-driven microglia modulate stress resilience through BDNF-dependent neurogenesis. *Sci. Adv.* 7:eabb9888. 10.1126/sciadv.abb9888 33731342 PMC7968840

[B57] Zhang Y. Chen Q. Chen D. Zhao W. Wang H. Yang M. (2021). SerpinA3N attenuates ischemic stroke injury by reducing apoptosis and neuroinflammation. *CNS Neurosci. Therapeut.* 28 566–579. 10.1111/cns.13776 34897996 PMC8928918

[B58] Zhang Z. Li D. Xu L. Li H. P. (2019). Sirt1 improves functional recovery by regulating autophagy of astrocyte and neuron after brain injury. *Brain Res. Bull.* 150 42–49. 10.1016/j.brainresbull.2019.05.005 31102754

[B59] Zhang Z. Zhang X. Wu X. Zhang Y. Lu J. Li D. (2022). Sirt1 attenuates astrocyte activation via modulating Dnajb1 and chaperone-mediated autophagy after closed head injury. *Cereb. Cortex* 32 5191–5205. 10.1093/cercor/bhac007 35106540

