# Supplementary material for: HDAC7 knockout mitigates astrocyte reactivity and neuroinflammation via the IRF3/cGAS/STING signaling pathway
Source: Front Cell Neurosci. 2025 Oct 28;19:1683595. doi: 10.3389/fncel.2025.1683595 (PMC12602527; doi:10.3389/fncel.2025.1683595)
Supplement: Supplementary file 1 [file Data_Sheet_1.pdf]

Supplementary materials

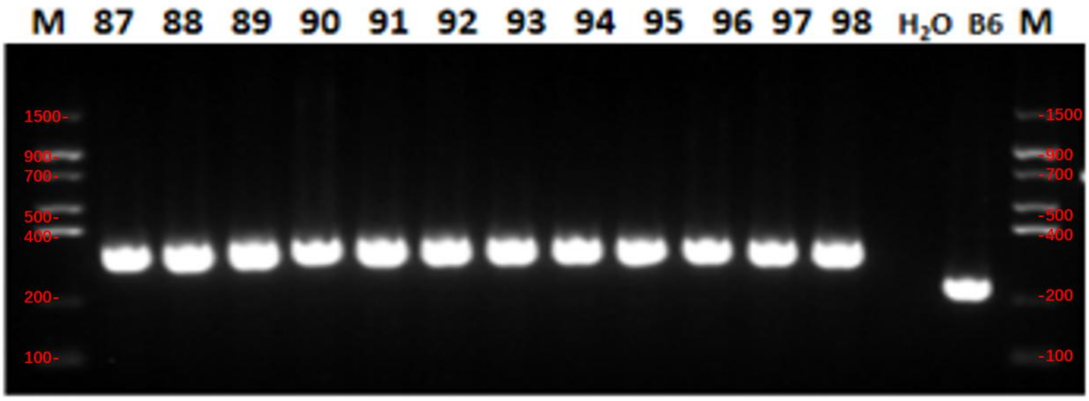

SFig.1 Schematic of Genotype Identification

Tail biopsies (0.5 cm) were collected from 14-day-old postnatal mice for genotyping to confirm successful generation of HDAC7-flox homozygous mice. Numbers 87–98 represent the ear tag identifiers of HDAC7-flox homozygous positive mice, with "B6" denoting the C57BL/6 background strain. In the agarose gel electrophoresis assay, water was used as a negative control to rule out exogenous DNA contamination.

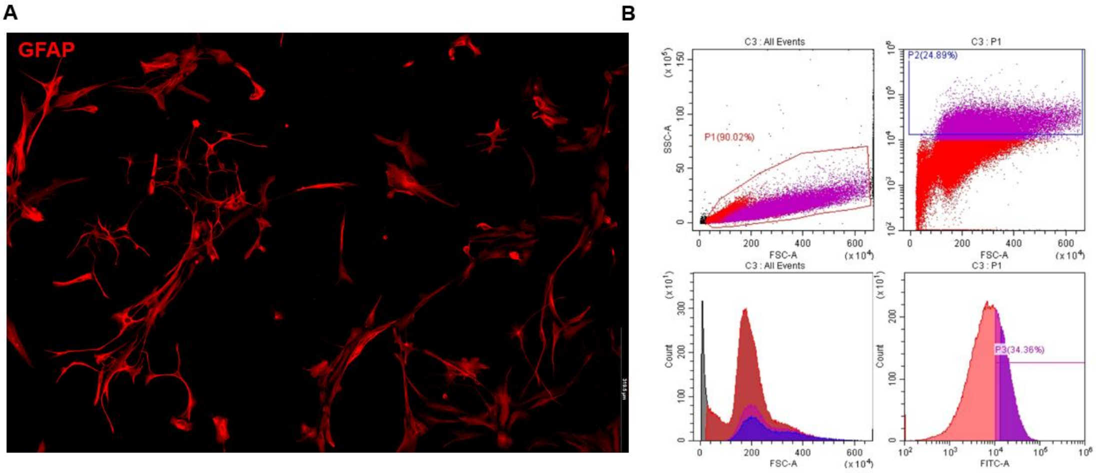

SFig.2 Assessment of astrocyte purity

A Representative immunofluorescence images of cultured primary astrocytes, scale bar is present in the images.

B Quantitative analysis of GFAP-positive cells in primary astrocyte cultures

Fig. 2

Name	Western Blot Figure
HDAC7	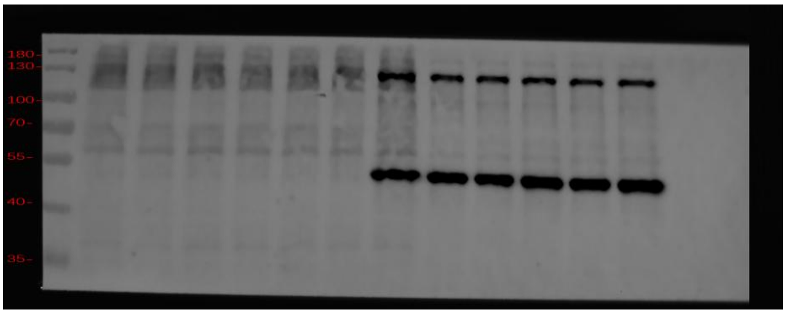
β-actin	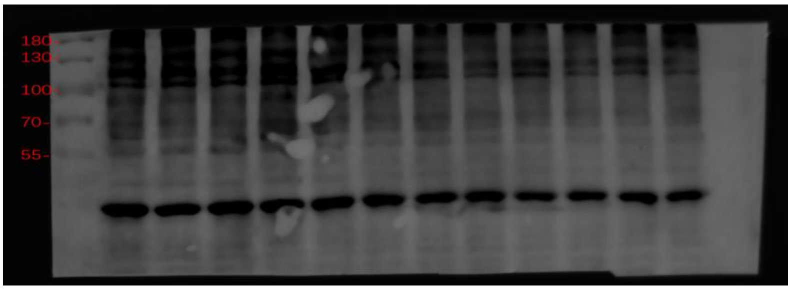
IL-6	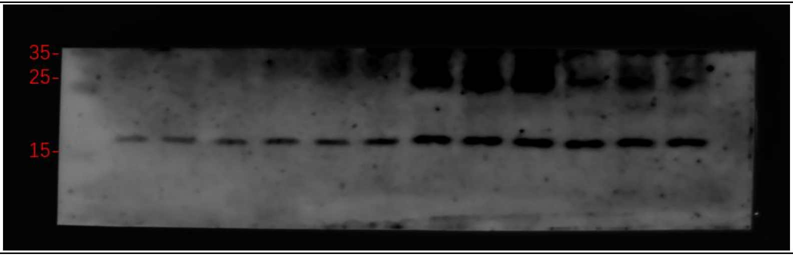
IL-1β	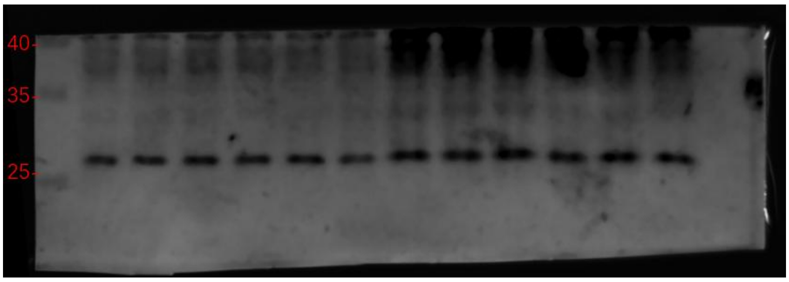

iNOS	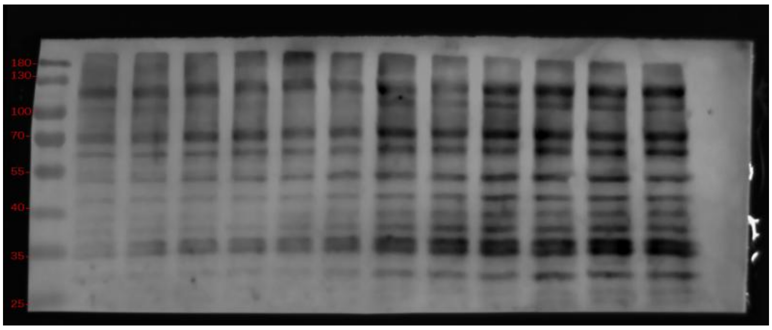
COX-2	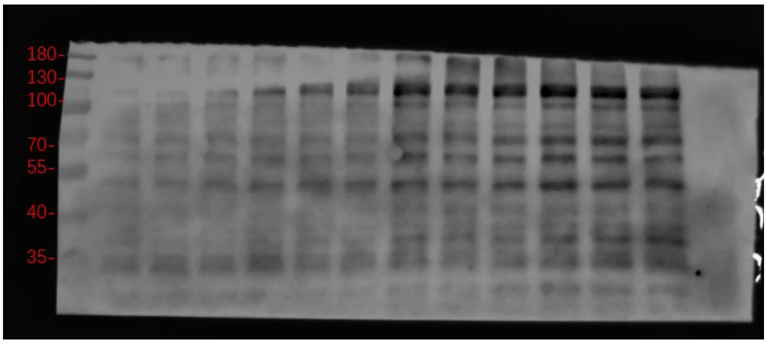
GFAP	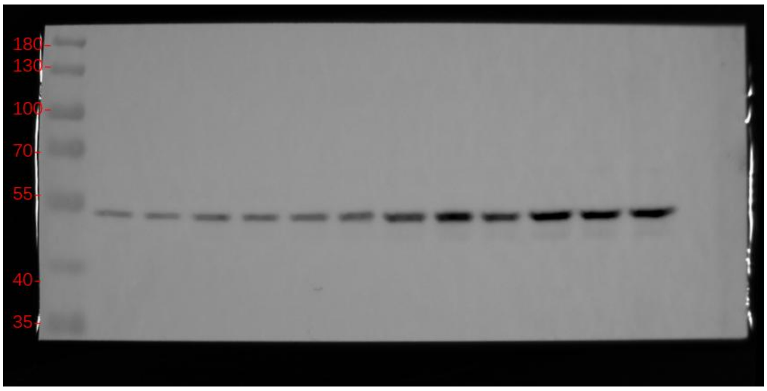
β-actin	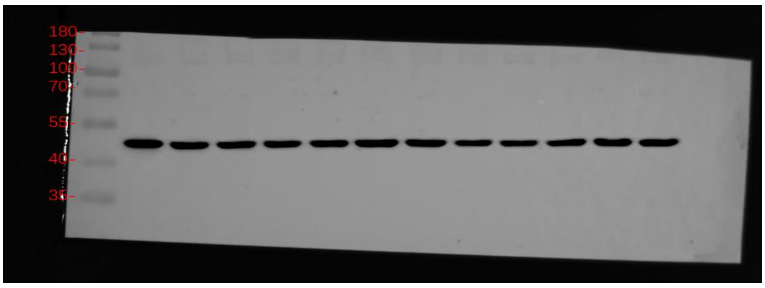
IRF3	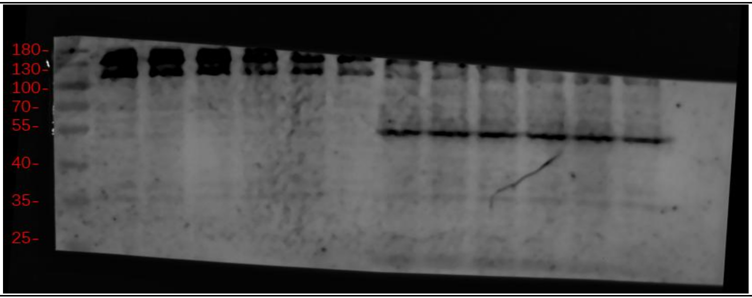

<table>
<tr><td>cGAS</td><td>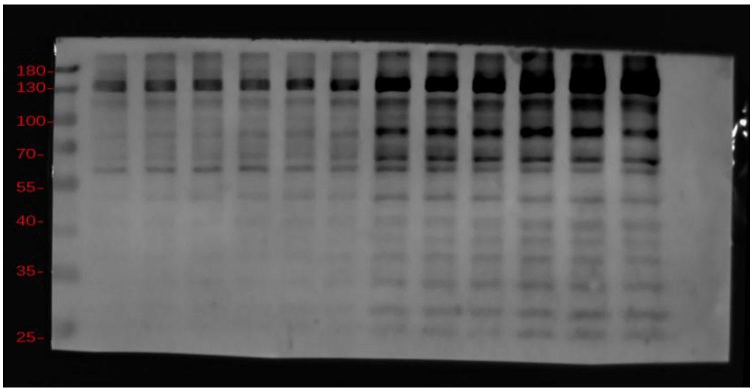
</td></tr>
<tr><td>STING</td><td>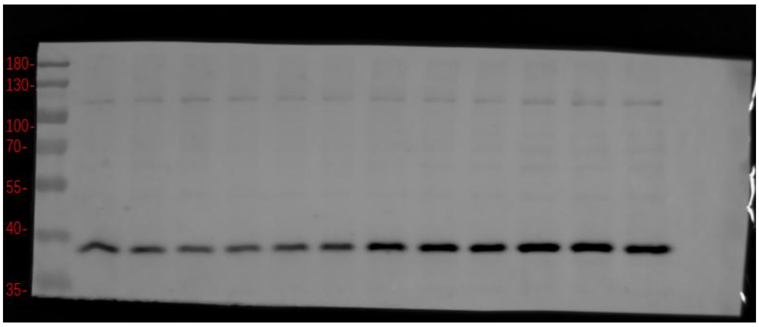
</td></tr>
<tr><td>β-actin</td><td>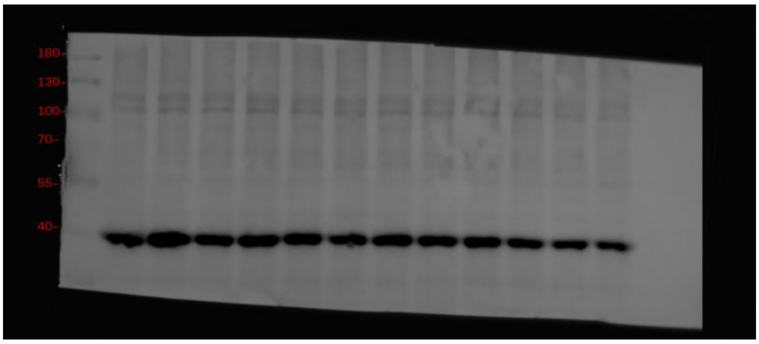
</td></tr>
</table>

Fig. 3

<table>
<tr><th>Name</th><th>Western Blot Figure</th></tr>
<tr><td>HDAC7</td><td>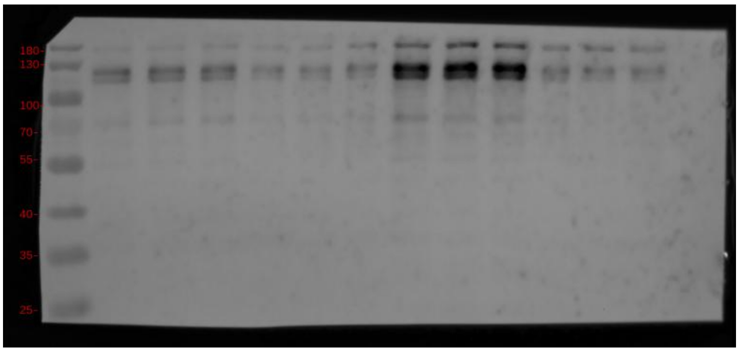
</td></tr>
</table>

β-actin	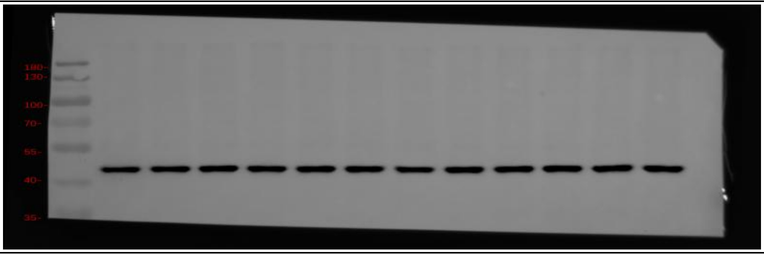
IL-6	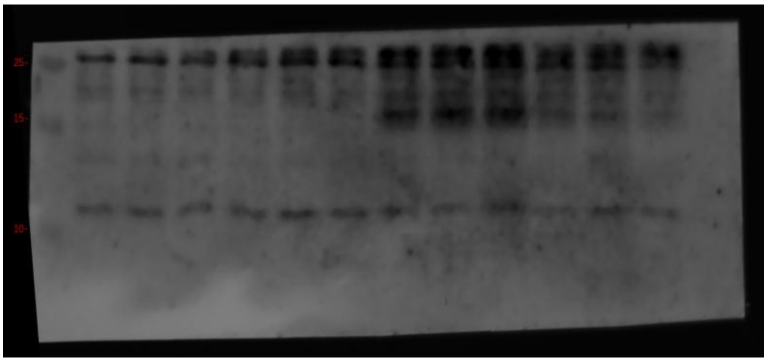
IL-1β	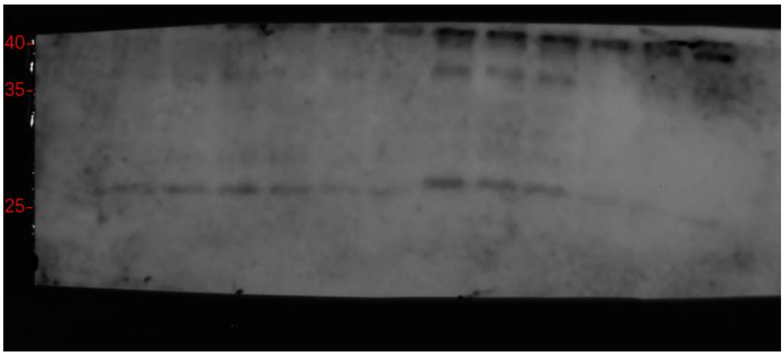
iNOS	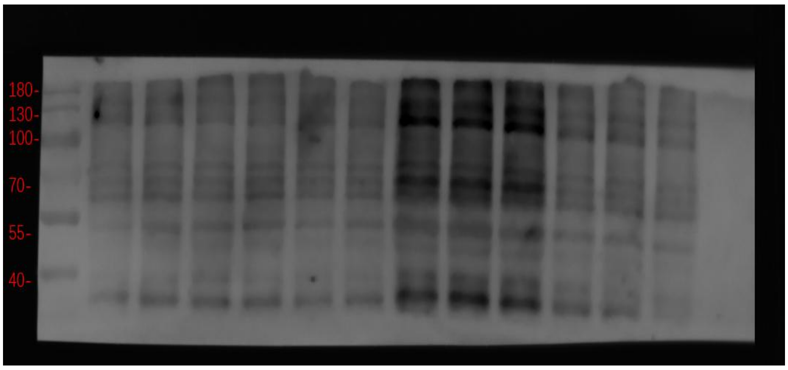
COX-2	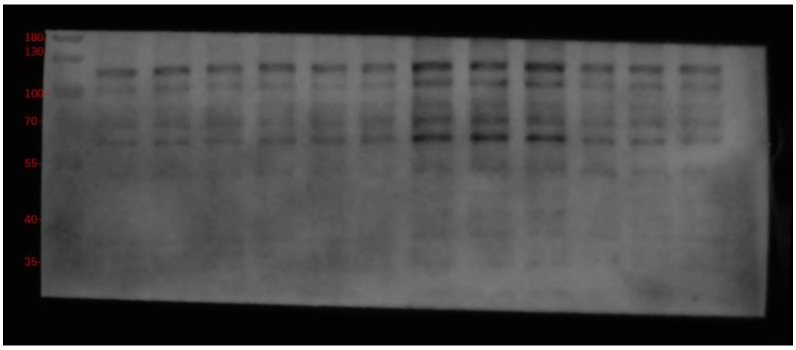

GFAP	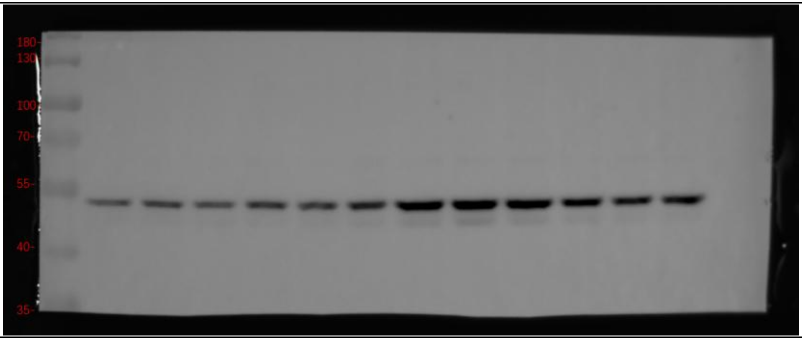
β-actin	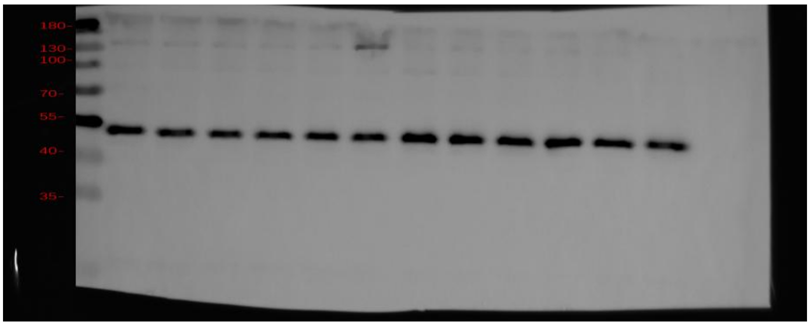
IRF3	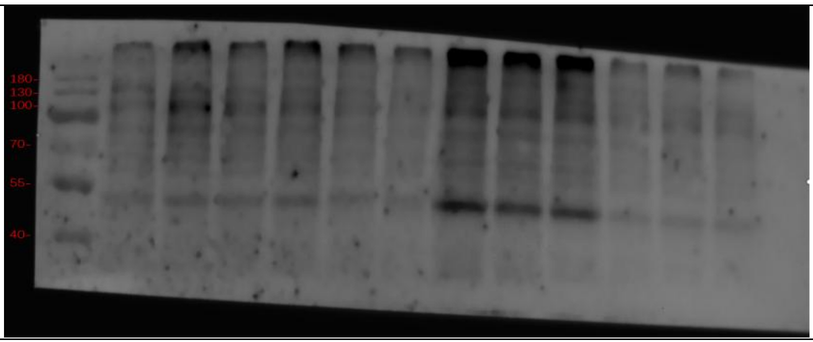
cGAS	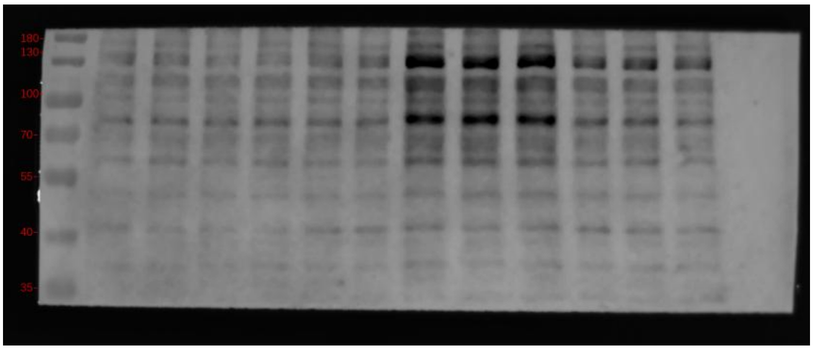
STING	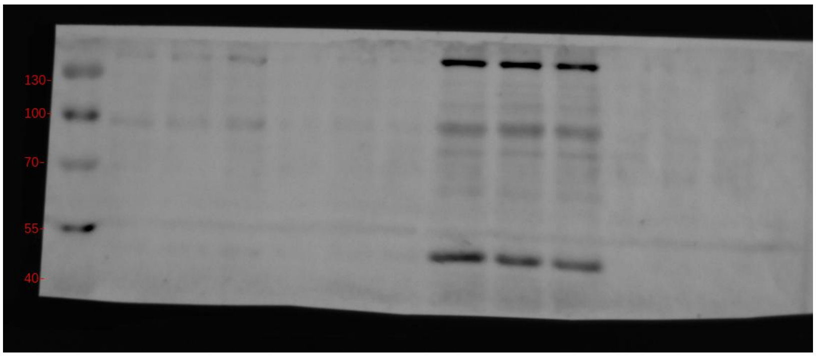

β-actin	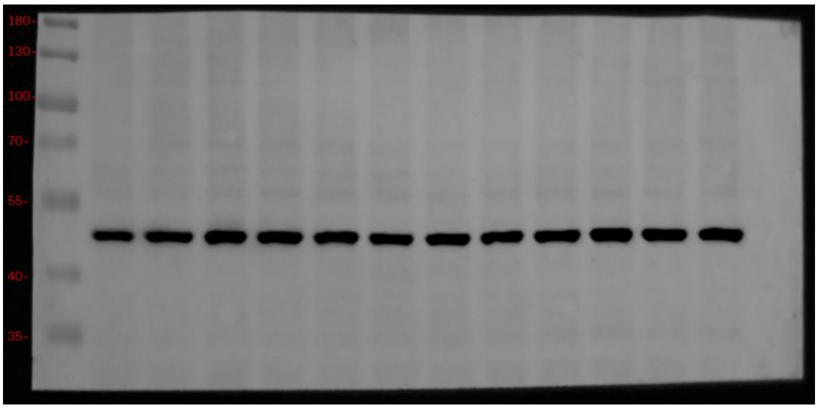

Fig. 4

Name	Western Blot Figure
IL-6	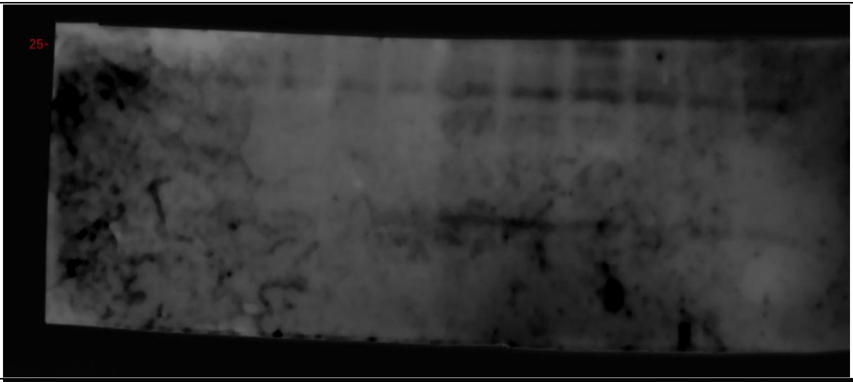
IL-1β	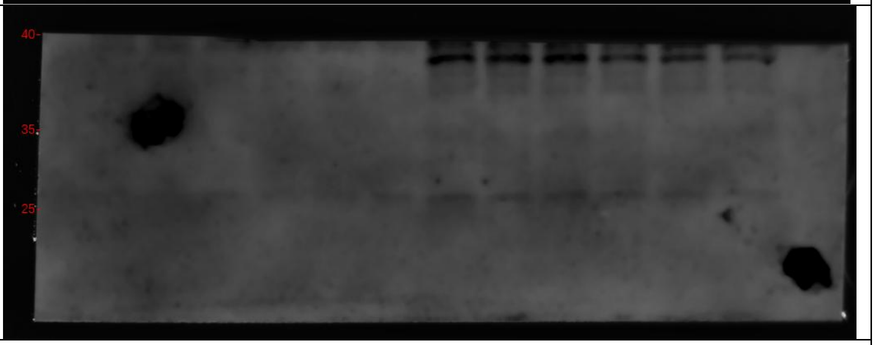
iNOS	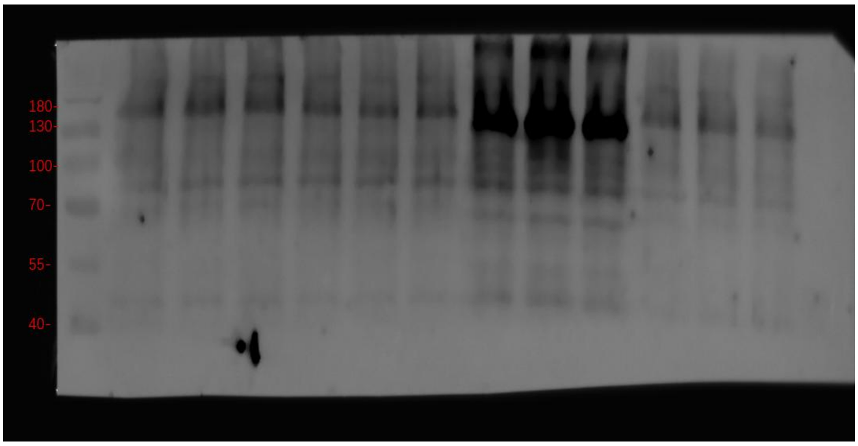
COX-2	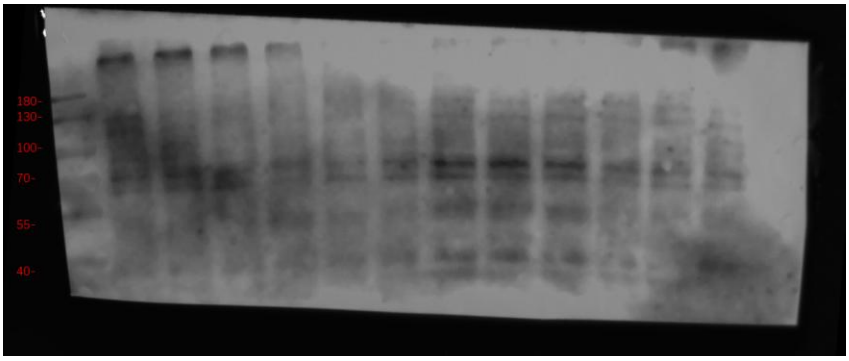

<table>
<tr><td>GFAP</td><td>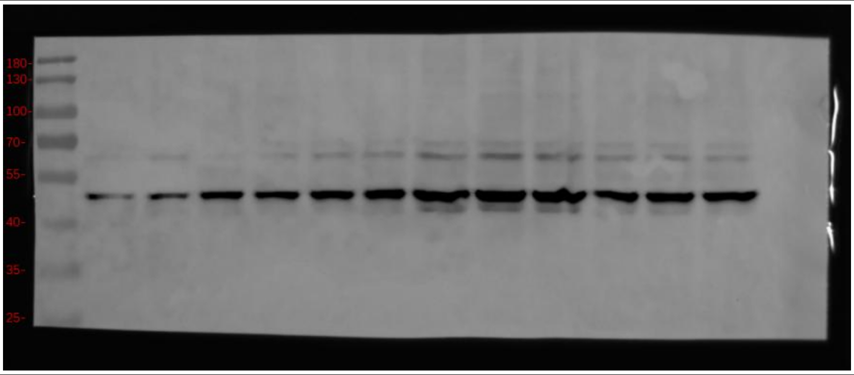
</td></tr>
<tr><td>IRF3</td><td>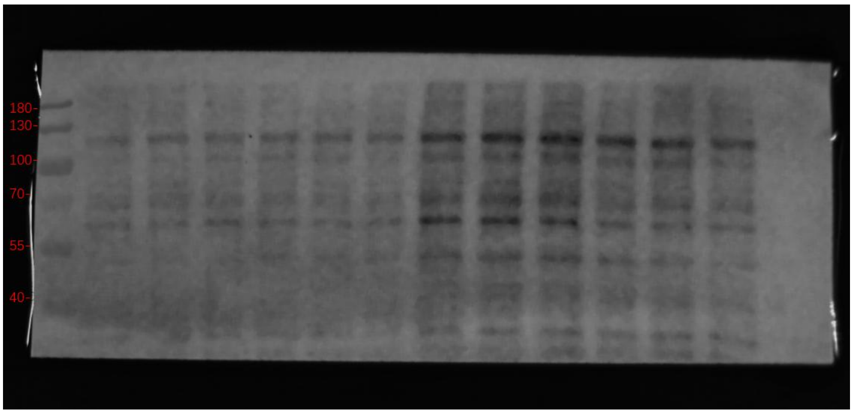
</td></tr>
<tr><td>cGAS</td><td>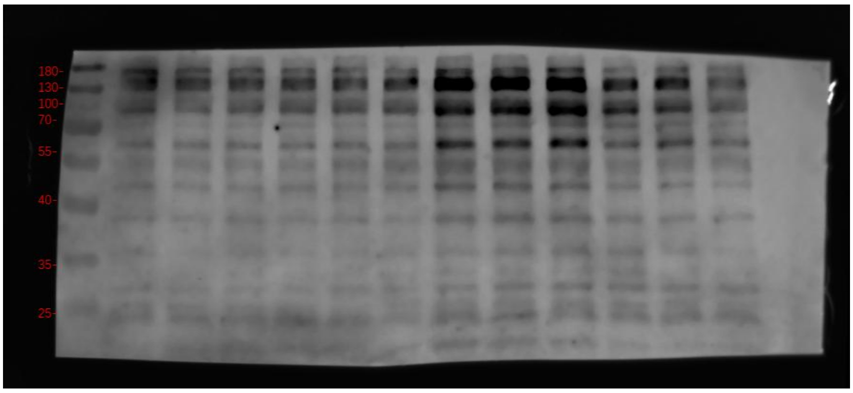
</td></tr>
<tr><td>β-actin</td><td>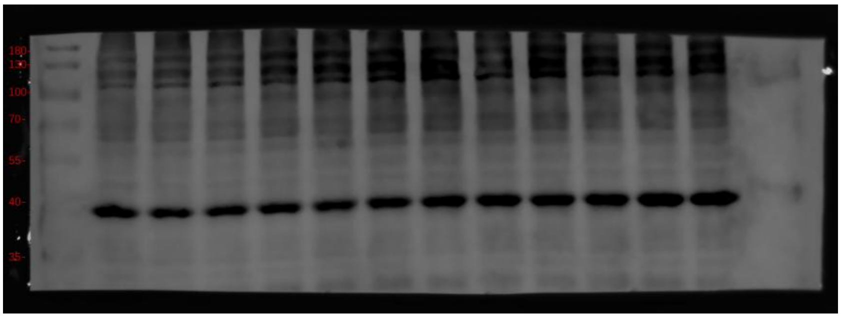
</td></tr>
</table>
